# Beyond emissions: unravelling the effects of ecosystem change on contaminant concentrations in herring from the Baltic Sea

**DOI:** 10.1007/s11356-025-36988-y

**Published:** 2025-10-02

**Authors:** Francesco Masnadi, John Martin Taylor, Johan Näslund, Elisabeth Nyberg, Andrius Garbaras, Elena Gorokhova, Agnes M.L. Karlson

**Affiliations:** 1 Department of Ecology, Environment and Plant Sciences, Stockholm University, Stockholm, Sweden; 2 Swedish Environmental Protection Agency, Stockholm, Sweden; 3 Center for Physical Sciences and Technology, Vilnius, Lithuania; 4 Department of Environmental Science, Stockholm University, Stockholm, Sweden; 5 Stockholm University Baltic Sea Centre, Stockholm, Sweden

**Keywords:** Altered productivity base, Biodilution, Changing ecosystem, *Clupea harengus*, Contaminant burden, Cyanobacterial bloom, Compound-specific isotope analyses, Trophic ecology

## Abstract

The effects of environmental changes on contaminant fate in the ecosystem are poorly understood, even in the otherwise well-studied Baltic Sea. This area is considered one of the most polluted in the world and is currently undergoing rapid shifts related to climate change and eutrophication. In this study, we focus on the effects of an altered productivity base and changes in food web structure on contaminant concentrations in the commercially important Baltic herring, which is also a key-species in the ecosystem. In herring of known size and age, collected within the Swedish National Monitoring Program for Contaminants in Marine Biota during the past two to three decades, retrospective analyses of contaminant concentrations and stable isotopes of carbon and nitrogen including amino acid-specific isotope analyses were performed. Partial least squares regression (PLSR) models were applied to dioxins, PCBs, and mercury time series to examine how biological, ecological, and environmental factors (i.e., age, trophic diversity and position, temperature, salinity, proxies of cyanobacterial blooms and ultimate nutrient sources, abundance of relevant benthic fauna as well as biomass and size structure of the zooplankton community) contribute in explaining contaminant concentrations in herring, beyond atmospheric deposition (the main contaminant input in the Central Baltic basin). Our results emphasize that the contaminant burden in Baltic herring is significantly influenced by factors other than atmospheric deposition. Primarily, changes in herring’s trophic ecology, together with nitrogen-fixing cyanobacterial blooms (supporting both growth biodilution and bloom-induced dilution), were linked to dioxin, PCB, and mercury concentrations in fish. Our results support the need to consider all potential ecological synergies and linkages when managing a rapidly changing system such as the Baltic Sea, in order to minimize noxious blooms without compromising the positive impact on contaminant concentrations in fish.

**Supplementary information:**

The online version contains supplementary material available at 10.1007/s11356-025-36988-y.

## Introduction

Coastal seas experience the effects of climate change and intensified anthropogenic activities worldwide, with negative effects on biodiversity and ecosystem functioning (Kannan and James 2009; Grimm et al. 2013). However, very little is known about the interactions between climate change, food web changes, and contaminant cycling on the contaminant concentrations in biota and hence human exposure to contaminants (Borgå et al. 2010; Dijkstra et al. 2013; Alava et al. 2017; Ek et al. 2021). In the well-studied Baltic Sea, one of the most intensely studied coastal areas with high data density and many long-term data series (Reusch et al. 2018), biota contain elevated levels of priority contaminants of concern such as polychlorinated dibenzo-p-dioxins (PCDDs) and polychlorinated dibenzofurans (PCDFs), polychlorinated biphenyls (PCBs) (Stockholm Convention; Bilcke, (2002)), and mercury (Minamata Convention; UNEP, (2013)). Contaminant concentrations in the Baltic Sea are generally high, largely due to a relatively dense population within the watershed (ca 85 million inhabitants), historical pollution loading, and a long hydrological residence time of around 30 years (Sinkkonen and Paasivirta 2000; Åberg et al. 2008; HELCOM 2023). Atmospheric emissions and the subsequent deposition, agricultural runoff, urban wastewater, and industrial discharges are primary contributors to contaminant loading in the Baltic Sea. Among them, atmospheric deposition has been identified as the main diffuse depositional pathway of contaminants in the Baltic Sea, primarily due to the extensive transportation of pollutants through the air that allows contaminants to travel over long distances and deposit into the sea (Assefa et al. 2019; Wagner et al. 2019). Additionally, sediments act as reservoirs, posing risks of re-release into the aquatic environment (Assefa et al. 2014; Kuliński et al. 2022). Despite the remarkable decline in direct emissions resulting from various management efforts including abatement measures, economic contraction, and industrial restructuring that took place in the HELCOM countries, contaminant concentrations in Baltic herring, a key species of commercial and ecological relevance, have remained relatively stable since the mid-to-late 1990s (Quaß et al. 2004; Miller et al. 2013; Elmgren et al. 2015; Assefa et al. 2019). This is problematic in many countries around the Baltic Sea, where fish products still contribute significantly to dietary contaminant intake, and Baltic herring in some regions continues to exceed the European Commission’s limits for dioxins and dioxin-like PCBs in food (Assefa et al. 2019; Fechner et al. 2023). The sale of Baltic Sea herring is allowed to domestic markets in Sweden and Finland, with dietary recommendations provided to consumers (EU Regulation (2011)), while sales have been banned in most EU Member States since 2002. Since then, the reduction in the concentration of harmful contaminants in fish has become an issue of primary concern, and Baltic herring has been identified as one of the key indicator species for contaminant monitoring within the Baltic Monitoring Program (BMP) (HELCOM 2004). Given herring’s central role in the food web and its high contaminant burden, bioaccumulation and biomagnification in this species pose an ecotoxicological risk to both marine ecosystems and human health (Wiberg et al. 2013).

Direct effects of climate change in the Baltic Sea include alterations in hydroclimate forces, i.e., temperature increases and pronounced multidecadal variability in salinity (Meier et al. 2022). These abiotic factors have been identified as the major drivers behind the Baltic Proper regime shift that occurred during the late 1980 s and early 1990 s which led to changes in the zooplankton community (Möllmann et al. 2009), in turn impacting herring growth as well as the uptake and bioaccumulation of contaminants throughout the food web (Peltonen et al. 2007). In addition, indirect effects of climate change include an altered productivity base such as larger blooms of nitrogen-fixing cyanobacteria (Kahru and Elmgren 2014), which comprise a feedback loop associated with eutrophication, water stratification, anoxic bottoms, and phosphorous release (Andersen et al. 2017; Murray et al. 2019). The altered production base can significantly impact the dynamics of contaminant distribution through the food web, potentially enhancing either biomagnification and/or biodilution. An increase in cyanobacterial blooms can induce a shift towards additional trophic levels—a process known as trophic lengthening (Steinkopf et al. 2024)—which could theoretically enhance biomagnification. In the northern Baltic Sea, increased terrestrial runoff from precipitation (i.e., brownification) has, in a similar way, been suggested to lead to an increase in trophic levels through an enhanced microbial loop and hence increase the biomagnification of mercury (Jonsson et al. 2017). Also, mercury methylation occurs under oxygen-deficient and anoxic conditions (Capo et al. 2022), and anoxic areas are also expected to increase in the future, especially in the Baltic Proper (Almroth-Rosell et al. 2021).

On the other hand, higher primary production—i.e., high phytoplankton densities—and subsequent organic matter sedimentation may enhance contaminant sequestration in the sediment (Shi et al. 2017). Specifically, the increased uptake of dissolved contaminants by phytoplankton cells leads to decreased contaminant concentrations in the water phase (i.e., phytoabsorption). The sinking of non-consumed organic matter (e.g., many dead cyanobacteria cells after the bloom; Lignell 1993), along with the contaminants they have absorbed, results in the removal of contaminants from the pelagic ecosystem through sedimentation (i.e., sediment traps; Turner 2002). Phytoplankton bloom events therefore cause not only sedimentation but also a so-called eutrophication-induced dilution of contaminants (Shi et al. 2017). Resultingly, a larger mass of phytoplankton may reduce the mass-specific burden of contaminants by phytoplankton cells which can cascade through the food web leading to lower contaminant levels in higher trophic levels, like fish (i.e., bloom-induced dilution; Chen and Folt 2005; Pickhardt et al. 2002). As a final mechanism, increased growth conditions in fish stimulated by increased primary and secondary production of N-fixing cyanobacteria (Hansson et al. 2007; Karlson et al. 2015; Svedén et al. 2016) can outpace the rate of contaminant accumulation, effectively diluting contaminants in tissue (i.e., somatic growth dilution; Simoneau et al. 2005).

Dietary uptake is the primary route of bioaccumulation and biomagnification of highly persistent contaminants throughout the trophic chain (Opperhuizen and Wijbenga 1984; Kiljunen et al. 2007; Ali and Khan 2019). Therefore, changes in prey availability may affect exposure to various contaminants. Shifts in the food web structure due to environmental changes have been shown to affect the abundance and quality of the zooplankton community relevant to the herring diet (Casini et al. 2010), together with increased inter-specific competition over prey with sprat and stickleback (Casini et al. 2010; Olin et al. 2022). The general decrease in growth of herring in the central Baltic over the last decades, due to the regime shift in the Baltic in the late 1980 s (Möllmann et al. 2009; Casini et al. 2010; ICES 2023), seems to be one of the most important factors counteracting the reduction of contaminants in herring expected from the ongoing emission reduction management framework (Wiberg et al. 2013). In fact, a longer time at sea (higher age) to reach a certain size, a characteristic of slow-growing individuals, will increase exposure to contaminants during the lifespan compared to faster-growing individuals (Peltonen et al. 2007). On the other hand, even if summer bloom-forming cyanobacteria are one of the most visually negative effects of climate change and eutrophication in the Baltic Sea (Karjalainen et al. 2007; Takolander et al. 2017; Viitasalo and Bonsdorff 2022), their role in promoting growth of high-quality prey and hence faster fish growth during summer when fish are food limited (Hansson et al. 2007; Karlson et al. 2015) could indirectly attenuate bioaccumulation of contaminants through somatic growth biodilution.

The time series of archived frozen material in the Environmental Specimen Bank (ESB) at the Swedish Museum of Natural History (SMNH) is one of the longest and most consistent datasets on contaminants in fish (Ammar et al. 2024). ESB-SMHI archived samples of marine benthos and fish have already been used to trace nutrient flows and to describe the trophic ecology of consumers (Ek et al. 2021; Karlson et al. 2024). In this study, retrospective stable isotope analyses were carried out on archived herring samples (both bulk carbon and nitrogen isotope—i.e., δ^13^C, δ^15^N—and amino-acid-δ^15^N measurements) to quantify changes in the diet via isotope niche metrics and trophic position calculations (Layman et al. 2007; Nielsen et al. 2015). Also, δ^15^N isotope values were used to trace cyanobacteria-derived nitrogen, thereby elucidation ultimate nitrogen sources (Motwani et al. 2018; Liénart et al. 2022).

**Fig. 1:**
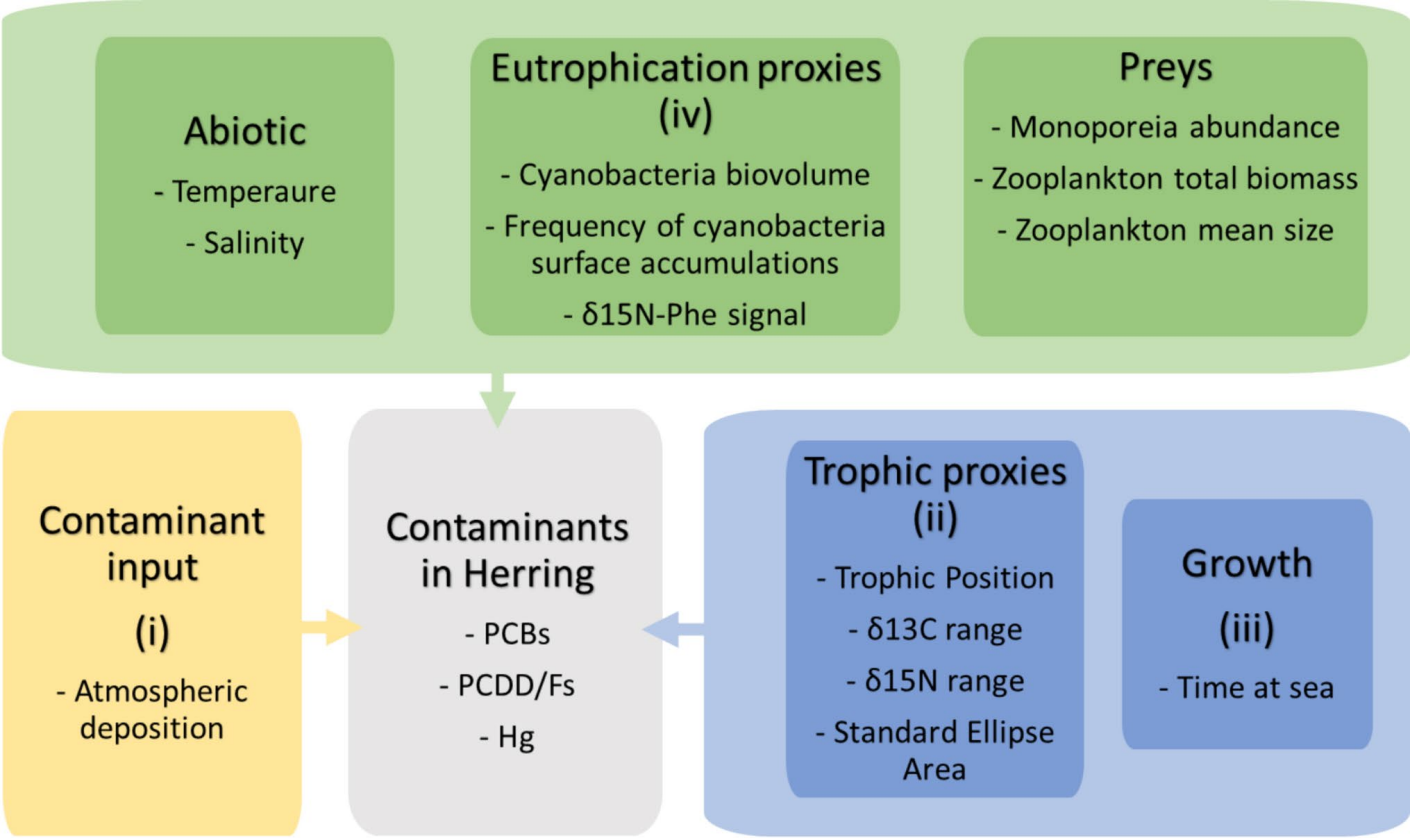
Graphical representation of Baltic Sea ecosystem dynamics taken into consideration for the analysis in this study and potentially affecting contaminant levels in herring. References to the hypotheses formulated in the aim of the study are in brackets

The main aim of this study was to better understand how some of the main biological, ecological, environmental, and anthropogenic pressures contribute to explaining contaminant concentrations in Baltic herring from the central Baltic Sea. First, we evaluated herring contaminant concentration time trends in relation to current and near-future safety thresholds. Secondly, we investigated how bottom-up factors related to changes in the production base can potentially affect concentrations of dioxins, PCBs, and mercury in herring. We expected that: (i) atmospheric emission/deposition is not the only driver for herring contaminant concentrations; (ii) changes in the diet and trophic niche of herring will influence the contaminant concentrations; (iii) longer time at sea required to reach a certain size (slower growth) counteracts the reduction of contaminants in herring; (iv) N-fixing cyanobacterial summer blooms reduce contaminant concentrations through biodilution. Expectations are graphically summarized in Fig. 1 together with potential predictors affecting contaminant levels in herring.

## Materials and methods

### Species and study area

**Fig. 2:**
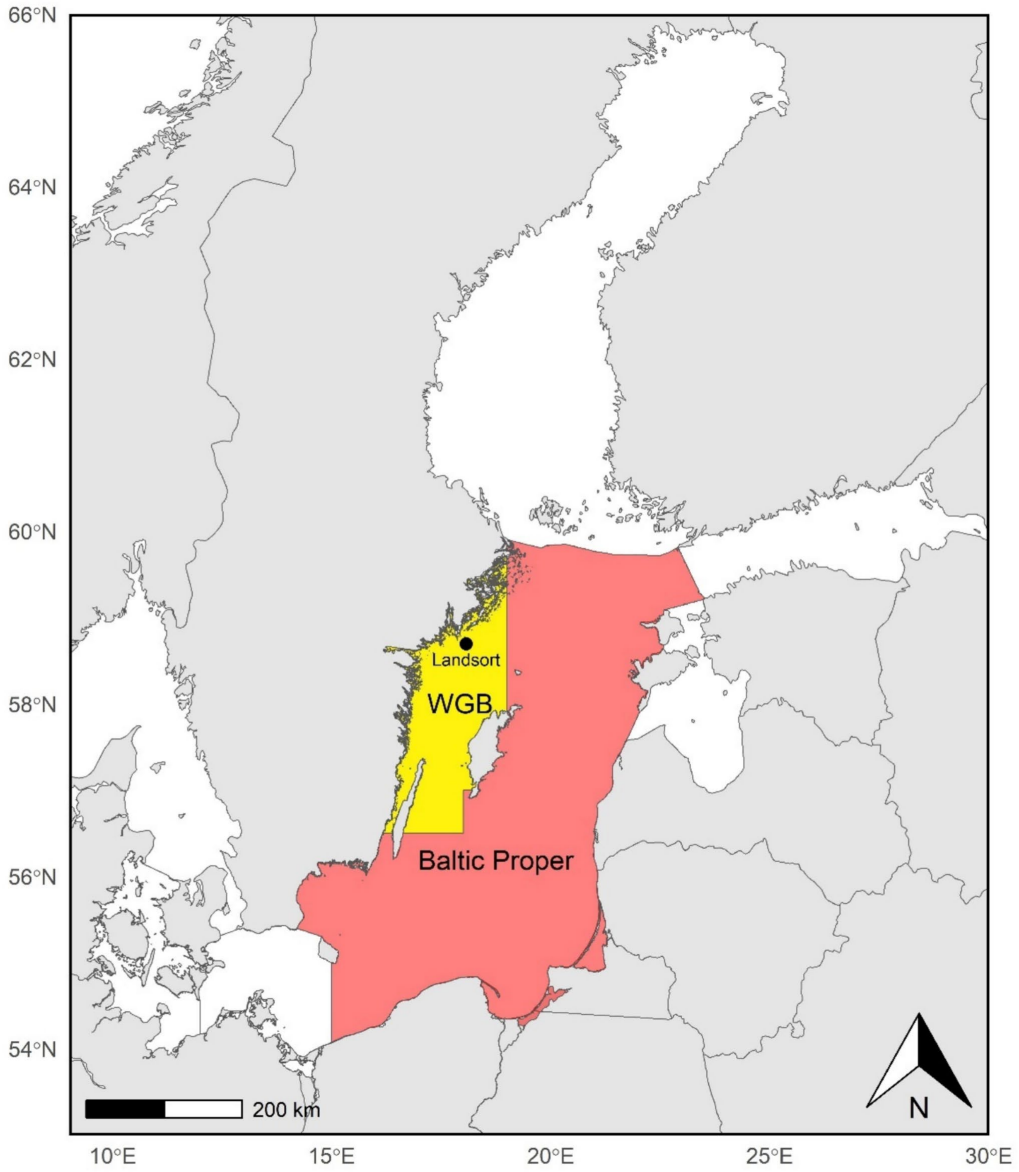
Map of the Baltic Sea with area of interest. The red area indicates the Baltic Proper while the yellow area represents the Wester Gotland Basin (WGB). Landsort sampling station is also shown for reference

The Baltic herring population includes several sub-populations of the Atlantic herring (*Clupea harengus*, Linnaeus, 1758) inhabiting the Baltic Sea, where they play a critical role in the Baltic Sea ecosystem. Herring are a pelagic migratory species that mainly feed on zooplankton and nektobenthos (Casini et al. 2004; Parmanne et al. 2006) and constitute a key food source of several marine species (from other piscivorous fish to seals and seabirds; Lundin 2011). In this study, we focused on herring within the Central Baltic Sea and specifically on data collected in the Western Gotland Basin (hereafter WGB; Fig. 2). In particular, the station Landsort (58° 42′N, 18° 04′E) was used as a proxy for the Central Baltic herring population since this is the station from the Swedish National Contaminant Monitoring Program for which samples stored at the Swedish Environmental Specimen Bank (ESB) were used for retrospective isotope analyses (Karlson et al. 2024). Since herring are highly mobile pelagic fish capable of several coastline-to-offshore migrations during their life cycle (Axenrot and Hansson 2004), we deem this station suitable for providing information on broader regional-level contamination conditions.

### Contaminant data

Within the Swedish National Monitoring Program for Contaminants in Marine Biota, herring of similar size and age (around 18 cm and 3–5 years old) were caught annually in October/November at different stations along the Baltic basin. As stated above, only Landsort data, which is the longest time series (1980–present), has been used in the study. For each individual fish, age (determined via scale reading), body length, lipid content, and contaminant concentrations were measured. CB-153 and Hg were measured in individual fish (20 in 1995–1996; 12 annually in 1997–2018). PCDD/Fs were analyzed in pooled samples (1 pool of 10–12 fish in 2005–2006; 2 pools of 12 fish annually from 2007 to 2018; see Table 1). Detailed information on collection and sample preparations can be found in Soerensen et al. (2024). Since similar-sized herring were analyzed, the age of the fish was used in statistical analyses as a proxy for growth rate. A longer time at sea to reach a certain size, characteristic of slow-growing individuals, would increase exposure to contaminants compared to faster-growing ones (Isosaari et al. 2006; Pandelova et al. 2008). The contaminants chosen for our analyses were selected following the toxicity level relevance of the chemical (included in the Water Framework Directive’s list of priority substances (EC 2000)) and availability of atmospheric deposition time series from the Baltic Sea Environment Fact Sheet (BSEFS) (HELCOM 2020). Specifically, the PCDD/Fs toxic equivalent value (pg TEQ/g lipid weight in herring muscle) of the seventeen 2,3,7,8-chlorinated congeners with toxicological relevance was selected as a proxy for dioxins and furans (PCDDs + PCDFs). Toxic equivalents (TEQs) were derived from individual congener concentrations using the 2005 toxic equivalency factors (WHO_2005_-TEFs; Van den Berg et al. 2006). Among the PCB congeners, CB-153 (μg/g lipid weight in herring muscle) was selected due to its long half-life and correlation with other congeners in biological matrices (Gladen et al. 1999; Ritter et al. 2011). Among the heavy metals, mercury (ng/g wet weight in herring muscle) was selected as the most relevant toxic and biomagnifying metal (UNEP (2013)). Dioxin and PCB concentrations, expressed in lipid weight (l.w.) in this study, were calculated by normalizing fresh weight concentrations to lipid content (i.e., standard procedures in the Swedish Monitoring Program), thereby accounting for lipid variability among individuals and years. Mercury, due to its strong affinity for protein, is reported in wet weight (also standard in the Swedish Monitoring Program). PCDD/Fs are formed as by-products in several industrial processes and combustion activities including wood burning, while PCBs have been commercially produced for use in household products since the 1920 s (Baars et al. 2004). Mercury (Hg) is predominantly anthropogenically generated (Burgess et al. 2013), and the highly toxic and bioaccumulative methylmercury was previously used as fungicides, or released as industrial by-products (Clarkson 1993). In this study, we present the sum of all PCDD/Fs instead of single congeners since the atmospheric deposition data available is provided as TEQ values (HELCOM 2020). However, the lower bound approach was used for the TEQ calculation (EU Regulation 2014), whereby values below the limit of detection (LOD) were set to zero (see Table S1 in Supplementary Information for congener-specific LOD frequencies). Hence, the TEQ results presented should be interpreted with some caution since differences in contribution to TEQ and bioaccumulative properties are known to vary between different congeners. For example, PeCDF2 is the most bioaccumulative among the furans while hepta- and octa-chlorinated PCDDs and PCDFs show limited uptake in herring due to excretion by feces (Isosaari et al. 2006). Still, TEQ values are easily comparable to previous work (e.g., Miller et al. 2013) and more meaningful when discussing food limit values and the combined toxic effects (EU Regulation 2014). In fact, TEQ provides a more accurate reflection of the potential health risks from exposure to a mixture of dioxins and furans, which often occur together in food sources. Current threshold values for environmental pollution hazard and human consumption for these three contaminant groups are presented in Table 1. The Baltic Proper (red area in Fig. 2) atmospheric deposition time series from HELCOM (2020) (based on the emission data) were used to represent annual contaminant input in the ecosystem as the main pollution pathway in the Baltic Sea (see the “Introduction” section).

**Table 1:** Summary table of collected data to be used as response variables in PLSR analyses

Contaminant	Threshold value	Sampling area/ period	Number of samples per year	Data sources
CB-153	EAC* = 1.6 μg/g l.w	Landsort (WGB) 1995–2018	1995–1996: 20 individual fish 1997–2018: 12 individual fish	ESB-SMNH^
WHO_2005_-TEQ PCDD/Fs	E** = 3.5 pg TEQ/g w.w Recalculated to 15 pg TEQ/g l.w. according to Soerensen and Faxneld 2022	Landsort (WGB) 2005–2018	2005–2006: 1 pooled sample of 10–12 fish 2007–2018: 2 pooled samples of 12 fish	ESB-SMNH^
Hg	EQS_biota_*** = 24 ng/g w.w. (methyl-Hg)	Landsort (WGB) 1995–2018	1995–1996: 20 individual fish 1997–2018: 12 individual fish	ESB-SMNH^

^*^EAC = Environmental Assessment Criteria (EAC), developed within (OSPAR Commission 2009)

^**^ EC = Maximum levels for certain contaminants in food (i.e., fishery product) (EC 2023)

^***^EQS_biota_ = Environmental Quality Standards in biota developed within the EC Directive 2008/105 (2008)

^ESB-SMNH = Swedish National Contaminant Monitoring Program data stored in the Environmental Specimen Bank (ESB) at the Swedish Museum of Natural History (SMNH)

### Stable isotope metrics

Fish analyzed for contaminants were also analyzed for stable isotopes (both bulk and amino-acid-δ^15^N measurements) as described in Liénart et al. (2021) and Karlson et al. (2024) respectively. Following bulk analyses of stable isotopes of carbon (δ^13^C) and nitrogen (δ^15^N), we calculated Layman metrics (i.e., δ^15^N range & δ^13^C range; Layman et al. 2007) and Bayesian standard ellipse area (SEA_B_, Jackson et al. 2011) using the SIBER package (version 2.1.9). These variables reflected trophic diversity and are used in this study as proxies to detect herring trophic ecology changes. Amino acid-specific δ^15^N analyses were performed on a composite sample of ten individuals (similar sized, 17–19 cm) to estimate trophic position (TP) using the equation described by Nielsen et al. (2015). Moreover, δ^15^N isotopic signature in Phenylalanine (hereafter δ^15^N-Phe) was used to trace the ultimate nitrogen source since this amino acid shows negligible fractionation compared to the bulk δ^15^N signal (McMahon et al. 2015; Liénart et al. 2022). Specifically, depleted (negative) δ^15^N-Phe signal is a good tracer of cyanobacteria-derived N via pelagic primary and secondary trophic pathways (Karlson et al. 2015; Liénart et al. 2022). Therefore, the inverse of the δ^15^N-Phe signal was used as a proxy of ultimate cyanobacteria-derived nitrogen, so that higher values indicate a higher cyanobacteria N-fixation contribution to fish growth. Bulk carbon and nitrogen isotope analyses were performed at the Center for Physical Science and Technology (Vilnius, Lithuania), using a Flash EA 1112 Series Elemental Analyzer connected to an Isotope Ratio Mass Spectrometer (DeltaV Advantage, Thermo Fisher). Amino acid-specific isotopes were analyzed at the University of California Davis Stable Isotope Facility (UC Davis) using GC-combustion isotope ratio mass spectrometry (GC-C-IRMS) and the method described in Karlson et al. (2024).

### Environmental data

Environmental data included abiotic and biotic data from various monitoring programs. Temperature (°C) and salinity (psu) were calculated from Baltic Sea Physics Reanalysis data (Copernicus Marine Service, 10.48670/moi-00013) as the average of the trophogenic zone (first 30 m) of the 4 months preceding the fish sampling in the fall (June to September) for the entire WGB (yellow area in Fig. 2). Biovolume of N-fixing cyanobacteria (the three bloom-forming species: *Aphanizomenon *sp.,* Nodularia spumigena*, and *Dolichospermum* spp.; see species composition by station in Figure S2 of the in Supplementary Information), as well as the abundance of nektobenthic fauna (i.e., the lipid-rich amphipod *Monoporeia affinis* which is an important prey for adult herring; Casini et al. 2004), was retrieved from the Swedish National Marine Monitoring Program available in the SHARK database (www.smhi.se). Zooplankton biomass (mg m^−3^) and mean size (μg ind^−1^) were calculated using biweekly data on mesozooplankton collected and analyzed following the standard protocol of the Baltic Sea Monitoring Program and aggregated as described in Gorokhova et al. (2016). *Monoporeia affinis* abundance and zooplankton biomass represent food availability, while zooplankton mean size is used as a proxy for prey food quality for zooplanktivorous fish (Casini et al. 2004; Gorokhova 2019). Predictors were computed as the average of the relevant station representing the WGB area (15 stations for *Monoporeia* and two stations for cyanobacteria and zooplankton, see Table 2 and Figure S3 in Supplementary Information for details). Station selection was carried out following the principle underlying the Karlson et al. (2020) and Liénart et al. (2021) studies. As for the abiotic data, cyanobacteria biovolume and zooplankton data time series were computed using the average summer monthly data (June to September), while *Monoporeia affinis* samplings were performed only in May. Moreover, the annual time series of frequency of cyanobacteria surface accumulations in the Baltic Proper (FCA, satellite data; red area in Fig. 2) from Kahru et al. (2020) was used as an additional proxy of cyanobacterial bloom intensity. These two cyanobacteria bloom proxies account for different aspects of the bloom phenomenon and are based on different types of data: satellite data for FCA (mainly captures the toxic species *N. spumigena*) and microscopy-based analysis for cyanobacteria biovolume in the water column (HELCOM 2018; Rahn et al. 2023). Moreover, while FCA values are based on wider basin-level high-frequency data, the biovolume estimate was retrieved from only two monitoring program stations, which are monitored twice a month. Day-to-day marine weather conditions at the sampling stations can have a large impact on the final estimated values as the extent of bloom can vary significantly over a few hundred meters distance or in the case of strong currents/rough seas.

**Table 2:** Summary table of collected data to be used as predictors in PLSR analyses

Category	Predictors	Proxy for	Rationale for use	Sampling area	Data sources
Contaminant inputs	Atmospheric deposition of CB-153, PCDD/Fs, Hg	Main contaminant input in the Baltic Sea	Main pollution pathway in the Baltic Sea	Whole Baltic Proper	HELCOM Baltic Sea Environment Fact Sheet (HELCOM 2020)
Growth	Time at Sea	Growth	A longer time spent at sea to reach a certain size may increase the bioaccumulation levels in fish	Landsort (WGB)	ESB-SMNH*
Trophic proxies	Trophic Position (TP)	Trophic/Diet	Changes in isotope niche metrics and trophic position can alter contaminant concentration (e.g., lower TP may mean feeding on prey with lower contaminant concentration)	Landsort (WGB)	Compound-specific isotope analysis (CSIA), DEEP** data from Karlson et al. 2024
	δ^13^C range (Layman metrics)	Trophic diversity		Landsort (WGB)	Stable isotopes from bulk, DEEP** data
	δ^15^N range (Layman metrics)	Trophic diversity		Landsort (WGB)	Stable isotopes from bulk, DEEP** data
	Standard Ellipse Area (SEA_B_)	Trophic niche		Landsort (WGB)	Stable isotopes from bulk, DEEP** data
Abiotic	Temperature (summer average)	Climate change	Alterations in environmental drivers may affect herring metabolism, feeding ecology and growth	Whole WGB (trophogenic zone)	Copernicus Marine Service data https://doi.org/10.48670/moi-00012
	Salinity (summer average)			Whole WGB (trophogenic zone)	Copernicus Marine Service data https://doi.org/10.48670/moi-00012
Eutrophication/altered productivity	δ^15^N-Phe signal in amino acids (inverse)	Cyanobacteria as a direct consequence of eutrophication, N-fixation contribution to food web	Depleted δ^15^N-Phe signal as a tracer of cyanobacteria-derived nitrogen	Landsort (WGB)	Compound-specific isotope analysis (CSIA), DEEP** data data from Karlson et al. 2024
	Cyanobacteria biovolume		Cyanobacterial blooms drive bloom-induced dilution and sustain the food web during summer (indirectly altering herring feeding ecology and growth)	Monitoring stations B1 & BY31 (WGB)	Sharkweb dataset https://sharkweb.smhi.se
	Frequency of cyanobacteria surface accumulations (FCA)			Whole Baltic Proper	Satellite data from Kahru et al. 2020
Preys	*Monoporeia* abundance	Prey availability and value	Lipid-rich amphipods are an important food source for adult herring	15 monitoring stations in Askö region (WGB)	Sharkweb dataset https://sharkweb.smhi.se
	Zooplankton total biomass		Main food source availability for zooplanktivorous fish	Monitoring stations B1 & BY31 (WGB)	Sharkweb dataset https://sharkweb.smhi.se
	Zooplankton mean size		Food value for zooplanktivorous fish	Monitoring stations B1 & BY31 (WGB)	Sharkweb dataset https://sharkweb.smhi.se

^*^ESB-SMNH = Swedish National Contaminant Monitoring Program data stored in the Environmental Specimen Bank (ESB) at the Swedish Museum of Natural History (SMNH)

^**^DEEP = Department of Ecology, Environment and Plant Sciences, Stockholm University

### Data pre-treatment and statistical analysis

Individual-level variables in herring, including contaminant concentrations, age, and isotope metrics (i.e., measurements derived from the Swedish National Monitoring Program for Contaminants in Marine Biota), were combined to obtain a single geometric mean value per year to align with standard procedures in environmental monitoring time series analyses (Miller et al. 2013; Assefa et al. 2019; Soerensen et al. 2024). The use of geometric mean reduces the influence of outliers (Caudill et al. 2007), ensures consistency with pooled PCDD/F data, and avoids pseudo-replication given that many predictor variables were only available at annual resolution. Individual-level variables in herring were missing in 2015. Additionally, values were missing from the isotope data for the years 1998, 2000, 2006, and 2013. Missing values were interpolated using the Stineman interpolation method to preserve the shape of the data (Stineman 1980). With regard to the CB-153 and Hg time series and the related statistical analyses, the range of all collected variables was reduced to the period 1995–2018 due to the limited temporal availability of some variables (i.e., abiotic variables from Copernicus, atmospheric deposition data, and isotopic metrics). Furthermore, the analyses concerning PCDD/Fs ranged from 2005 to 2018 due to the later start of the monitoring program for this group of contaminants in the area. Even though the monitoring program is designed to minimize the influence caused by intra-annual variability in the biological variables by selecting fish of similar size and age, a slight variation in fish body length (average total length TL = 17.7 cm, range = 15–19 cm) was found in the samples. The potential influence of TL as a confounding factor was therefore tested following the methodology by Bignert et al. (1993). Specifically, we assessed relationships between each individual-level variables in herring and TL using multiple linear regressions. Only the regression between time at sea (age) and TL was found to be statistically significant (*r*^*2*^ = 0.47, *p*-value < 0.05) and the variable was adjusted as follows:

1 $$Var_{adjusted}=Var_{measured}+A\ast\left(TL_{average}-TL_{measured}\right)$$

where *A* is the regression coefficient of the linear relationship and TL_average_ − TL_measured_ calculates how each sample’s TL differed from the mean of the time series (i.e., final values were estimated as if fish length was constant at the average value through the whole time series).

To check potential changes over time for all the variables collected (both starting from 1995 to 2005), we employed a single-breakpoint detection analysis in combination with a segment-specific linear model (Bai and Perron 2003). In summary, the breakpoint and segment-specific slopes were estimated by minimizing the residual sum of squares over all possible single‐break partitions. Each segment was classified as increasing or decreasing or no trend based on the directionality and statistical significance of the linear model (*p*-value < 0.05). Model selection for the presence and timing of a breakpoint was guided by the Bayesian Information Criterion (BIC). When no breakpoint was supported (i.e., the null model without a structural change yielded the lowest BIC), the entire series was treated as a single linear segment. Before that, all predictor variables were *Z*-score normalized (mean-centered and variance-standardized) to focus on the changes in relative rather than absolute values (Karlson et al. 2020).

Partial least square regression (PLSR; Wold et al. 2001) was used to investigate the contribution of 14 biological, ecological, environmental, and anthropogenic predictors to contaminant concentrations in herring: atmospheric deposition, time at sea (age), trophic position (TP), δ^13^C and δ^15^N range, SEA_B_, temperature, salinity, *Monoporeia* abundance, zooplankton biomass and mean size, cyanobacteria-derived nitrogen (δ^15^N-Phe signal), biovolume and FCA. Predictors were selected based on existing literature, with the rationale for their inclusion provided in Table 2. As response variables, CB-153, PCDD/Fs, and Hg concentrations were used in three separately fitted PLSR models. PLSR was selected as the most suitable analytical method based on the dataset’s high dimensionality (i.e., a large number of predictors). PLSR can extract latent variables (i.e., components) that capture the most relevant information from the predictors, thereby simplifying the model and improving its performance. To reduce the potential for spurious correlations arising from shared temporal trends, all variables were linearly detrended by applying Eq. 1 with “year” as the independent variable instead of fish body length. This approach is mathematically equivalent to extracting residuals from a simple linear regression of each variable against time. Although we recognize that some temporal trends may be nonlinear, linear detrending was applied as a first-order correction to minimize temporal autocorrelation and ensure that the associations detected in the PLSR models reflect relationships beyond those driven by time. This step is illustrated in Supplementary Information Figure S4. This approach allowed to improve the interpretability and robustness of the PLSR model, particularly when dealing with non-stationary time series data (Iler et al. 2017). Therefore, PLSR analyses were performed on year-detrended variables using the NIPALS (non-linear iterative partial least squares) algorithm and the leave-one-out cross-validation method to select the minimum number of components. All potentially relevant predictors were included in the initial models, and variable selection was performed backwards based on the importance of projection (VIP) scores (predictor is retained if VIP > 0.7). In addition, time-lag effects (i.e., the effect of the previous year’s predictor value on the observed contaminant concentration for a given year) were tested using cross-correlation analysis between response and predictor variables (max lag tested was 5 years considering the life span of the fish sampled). When a cross-correlation was significant (see Supplementary Information Tables S5 to S7), the lagged predictor was included as an additional predictor in models and retained if the model fit improved. The rationale for including predictors in the initial models is presented in Table 2. Lastly, even if PLSR allows for correlating predictors, multicollinearity among the predictors was evaluated using a correlation matrix plot. Model evaluation and validation were based on model explanatory (*R*^2^*Y*) and predictive capacity (*R*^2^*Q*). A model was considered good when *R*^2^*Y* > 0.7 and *R*^2^*Q* > 0.4 (Lundstedt et al. 1998). The assumption of independence of the observations was checked by testing the autocorrelation of model component residuals using the ARIMA algorithm (see Supplementary Information plots S8 to S10). This is particularly important in time series data where autocorrelation may be present. Since no significant autocorrelation was detected, no further action was taken. A predictor’s importance in the final models was ranked based on the absolute value of weighted regression coefficients (regression coefficients multiplied by the sum of squared weightings; WRC). WRC reflects the degree to which each predictor is associated with variation in the response variable (reflected by the strength and direction of the regression coefficients) by its overall contribution to the model’s components (reflected by the sum of squared weightings), yielding a combined measure of importance. Interpolation was computed using *imputeTS* R package (ver. 3.3, September 2022). Breakpoint detections were computed using the *strucchange* R package (ver. 1.5.4, September 2024) while multicollinearity was assessed by using the *PerformanceAnalytics* R package (ver. 2.0.4, October 2022). PLSR analyses were performed using the *pls* R package (ver. 2.8–3, November 2023; code provided at the end of Supplementary Information).

## Temporal changes

**Fig. 3:**
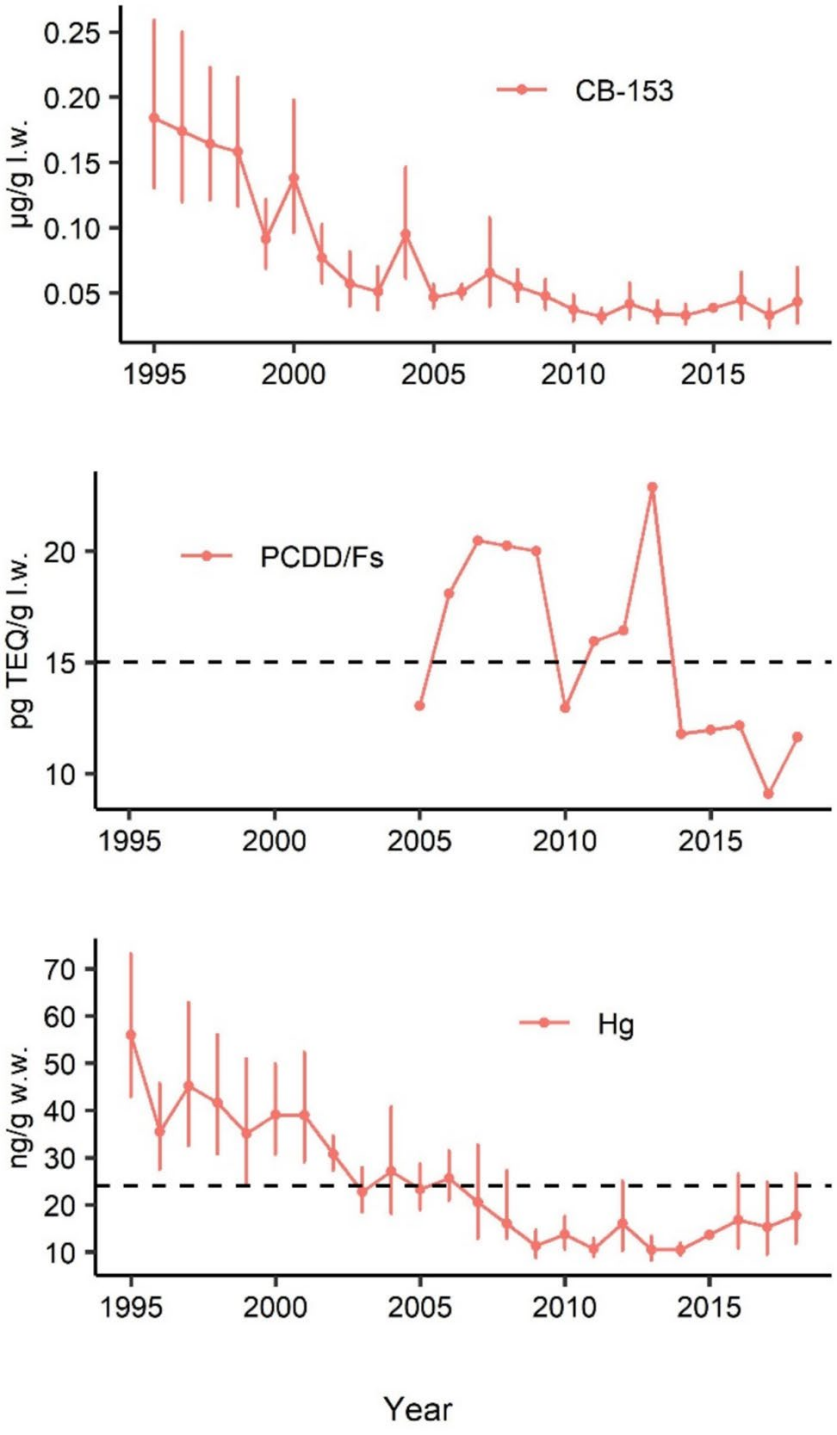
Temporal development of the contaminant concentration in herring from the WGB (Landsort station). The 95% confidence interval is shown for CB-153 and Hg where individual-level samples were available. Trend segments were identified through breakpoint analysis, fitting separate linear models to each identified segment (in this case, no breakpoint was detected). Red = decreasing significantly over time (*p*-value < 0.05). The dashed lines indicate the limit values according to relevant regulations (see Table 1). No limit is presented for CB-153 since out of plot scale (1.6 μg/g l.w.). l.w., lipid weight; w.w., wet weight

**Fig. 4:**
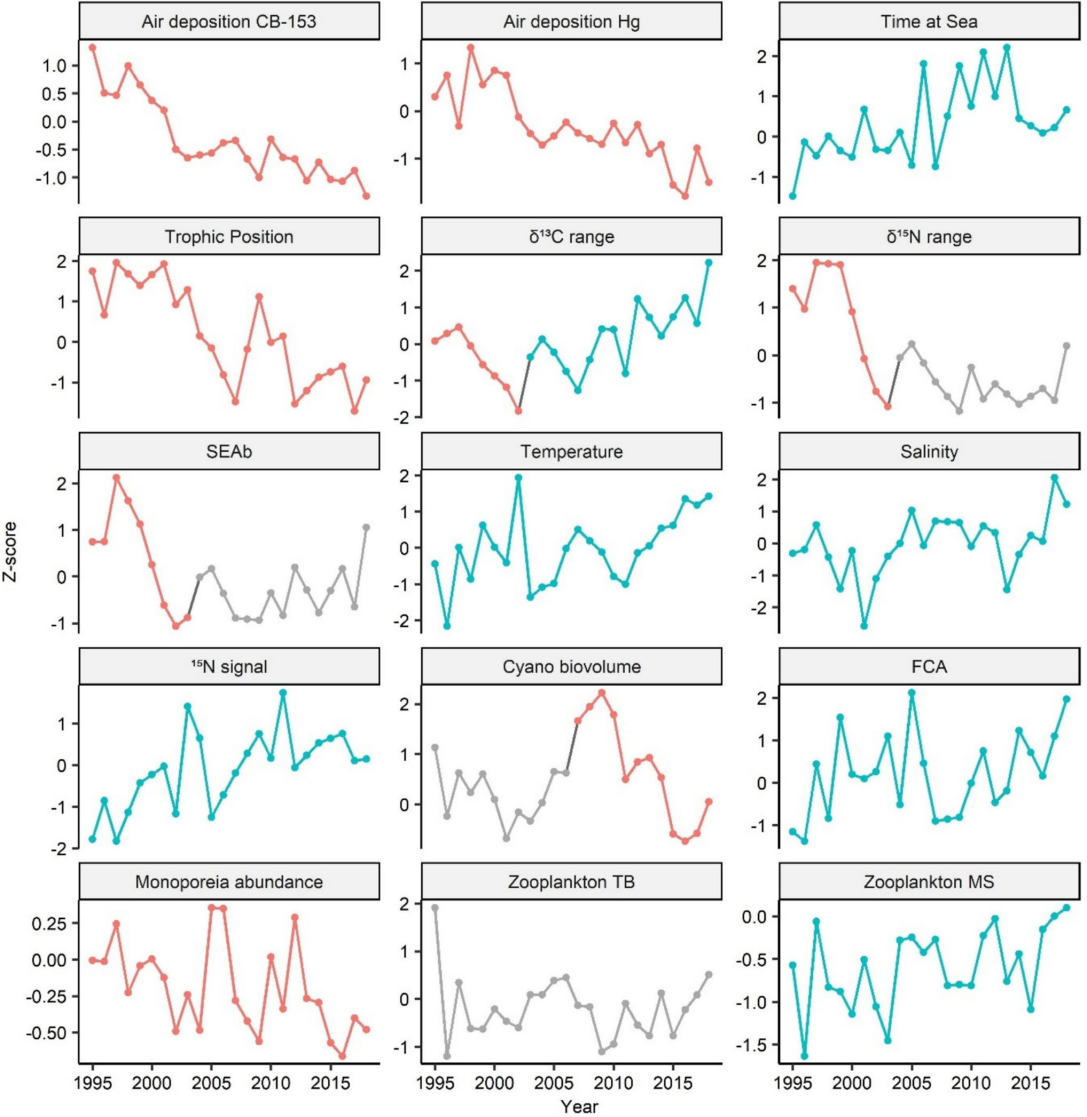
Temporal development of predictor variables selected for the CB-153 and Hg PLSR models (1995 onwards). Trend segments were identified through breakpoint analysis, fitting separate linear models to each identified segment. Colors represent the direction of significant temporal trends (*p*-value < 0.05): red indicates a significant decrease, blue indicates a significant increase, and grey denotes no significant change. Data shown are standardized (*Z*-score normalization). Detailed description of the predictors in Table 2

**Fig. 5:**
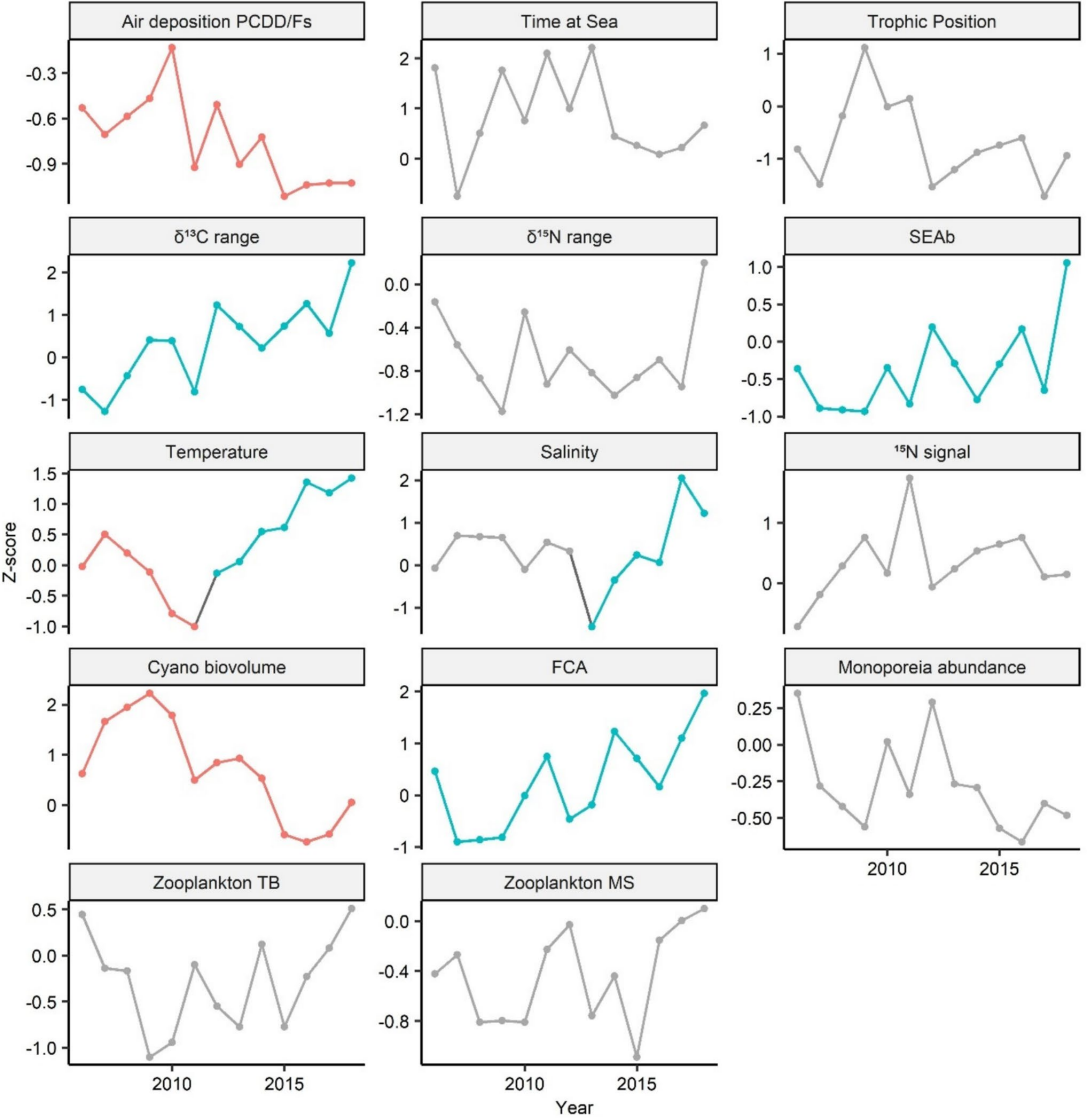
Temporal development of predictor variables selected for the PCDD/Fs PLSR models (2005 onwards). Trend segments were identified through breakpoint analysis, fitting separate linear models to each identified segment. Colors represent the direction of significant temporal trends (*p*-value < 0.05): red indicates a significant decrease, blue indicates a significant increase, and grey denotes no significant change. Data shown are standardized (*Z*-score normalization). Detailed description of the predictors in Table 2

The time series of contaminant concentrations in herring are shown in Fig. 3 and Table S11 and predictors in Fig. 4 (data from 1995) and Fig. 5 (data from 2005). Significant trends are indicated in the figures. We found significant downward trends for all three contaminant groups in fish. During the study period, CB-153 was always below the target level for environmental pollution hazards and human consumption, while mercury remained below the limit from the early 2000s. Although the dioxin time series is shorter, concentrations in more recent years have significantly decreased below the regulations’ limit (Fig. 3). Atmospheric deposition showed a significant decreasing trend for all the contaminants (Figs. 4 and 5). Starting from 1995, time at sea (proxy for herring growth) increased significantly over time while the trophic position decreased (Fig. 4). Instead, the three isotopes’ metrics (δ^15^C range, δ^13^C range, and SEA_B_) significantly decreased until the early 2000 s, after which they showed no consistent trend, except for the δ^13^C range, which exhibited a subsequent increase (Fig. 4). Considering the time period from 2005, only SEA_B_ and δ^13^C range increased while the other trophic predictors show no trend (Fig. 5). Among the prey predictors, *Monoporeia* abundance decreased while zooplankton mean size increased only from 1995 (Fig. 4). Both temperature and salinity showed a positive increase in the area from 1995 (Fig. 4), although when considering data from 2005, both showed an increasing trend only after 2010 (temperature decreased in the first few years; Fig. 5). The cyanobacteria-derived nitrogen (δ^15^N-Phe signal) and bloom intensity (FCA) showed increasing trends from 1995 (Fig. 4). Cyanobacteria biovolume showed a significant decreasing trend only starting from 2007 onward (Fig. 4) and confirmed in the shorter time period (Fig. 5).

### Linking environmental and ecological changes to contaminant concentrations in herring

**Fig. 6:**
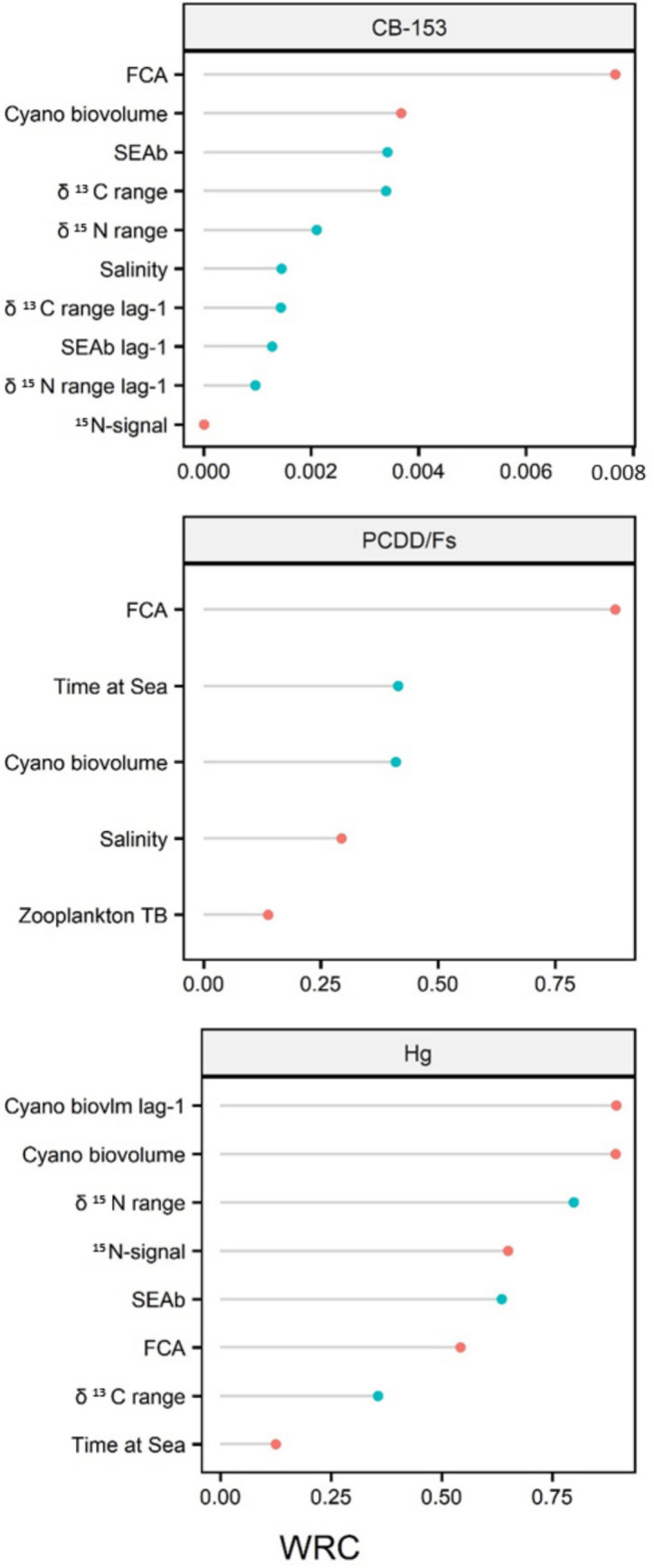
PLSR models result: impact of each predictor on the response variable. Predictors are ranked by importance based on the absolute value of weighted regression coefficient (WRC). Blue dots are predictors with a positive influence on the response variable, red dots with a negative influence. Detailed description of the predictors in Table 2

Correlation matrix plots (both considering time series from 1995 for CB-153 and Hg and 2005 for PCDD/Fs) did not show high correlations between the candidate predictors for PLSR analysis (pairwise correlation coefficients < 0.8, absolute value; see Supplementary Information Tables S12 and S13). PLSR models for CB-153 and Hg had adequate performance (*R*^2^*Y* = 0.84, *R*^2^*Q* = 0.65 and *R*^2^*Y* = 0.76, *R*^2^*Q* = 0.49 respectively; Table 3), while the PCDD/Fs model did not reach the minimum criteria (*R*^2^*Y* = 0.58, *R*^2^*Q* = 0.26; Table 3), probably due to the shortness of the series analyzed (2005–2018). Nevertheless, when compared with a random permuted model, the PCDD/Fs model performed significantly better (see Supplementary Information Figure S14 for a detailed description of the random permutation analyses). This indicates that, despite the lower predictive capacity, the model was still able to capture the underlying relationships between the predictors and the response variable. The effect direction and relative importance (WRC) of predictors in the three models are presented in Fig. 6. Atmospheric deposition was not found to be a relevant predictor in any of the models. Instead, the concentrations of contaminants in herring were mainly driven by a combination of trophic and cyanobacteria-related predictors. Within the trophic predictors, δ^15^N range, δ^13^C range, and SEA_B_ were the most important positively affecting CB-153 and mercury concentration in fish. Moreover, although of lesser importance, the values of the previous year had also similar effects (i.e., time-lag effects). No effect of trophic predictors was observed on PCDD/Fs concentration. All cyanobacteria/altered productivity base proxies had a highly important and negative association with CB-153 and mercury concentration (especially FCA for CB-153 and cyanobacteria biovolume and its time-lag effect for mercury). FCA was ranked higher in importance for PCDD/Fs with a negative effect, whereas cyanobacterial biovolume, in contrast, had a positive association on contaminant concentrations. Time at sea (age) had a positive effect on PCDD/Fs concentration in fish while negative but of minor importance for mercury. Oceanographic parameters, i.e., temperature and salinity, showed no or low importance as predictors with mixed effect direction. Among predictors related to prey availability and prey value, zooplankton total biomass was the only one showing a negative association with PCDD/Fs, albeit minimal.

**Table 3:**
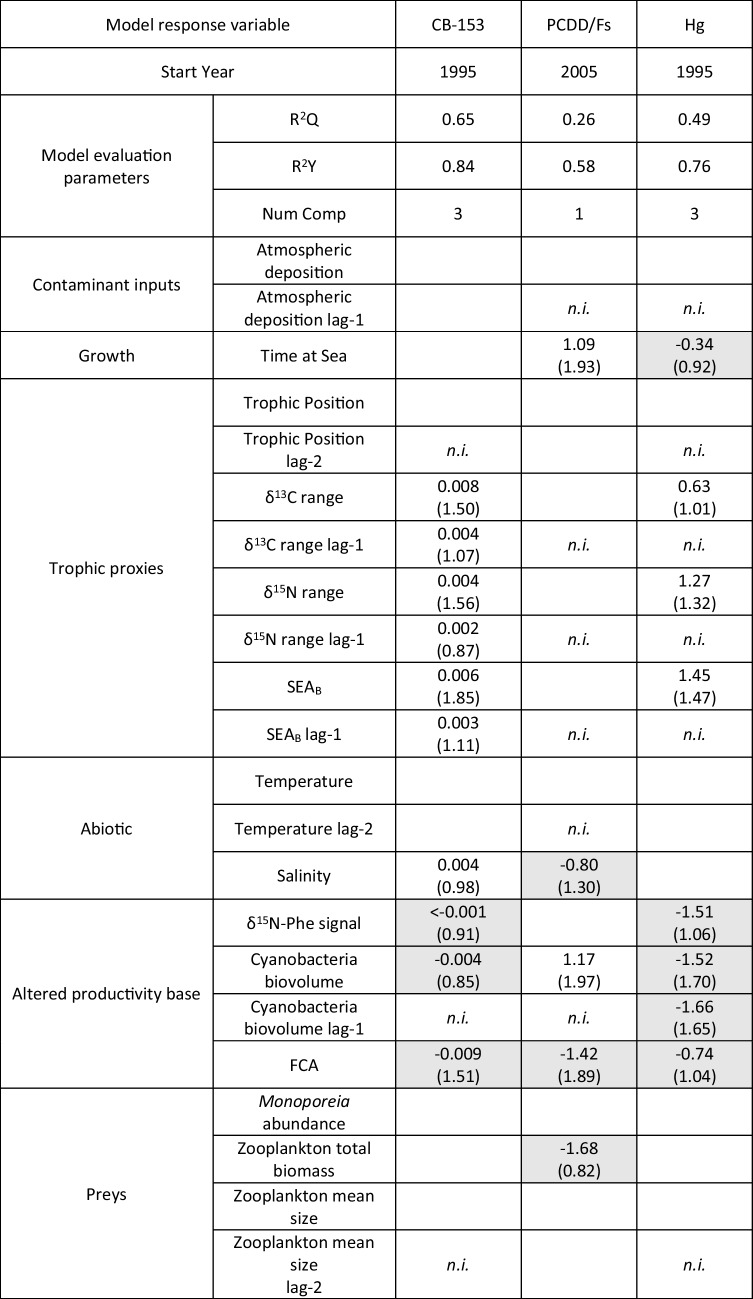
PLSR analyses results for the three contaminant groups tested. R^2^Y is the explanatory capacity coefficient, R^2^Q is the prediction capacity coefficient. The values are regression coefficients while numbers in brackets are the VIP scores. White cells are predictors with a positive influence on 1090 the response variable, shaded grey cells with a negative influence. Empty cells indicate that the predictor was included in the initial model but later excluded during the VIP-based variable selection process. *n.i.*: Lagged predictor not included in the initial model

## Discussion

The significant decrease in PCBs (i.e., CB-153), dioxins (i.e., PCDD/Fs), and mercury concentrations in herring tissue from the Landsort area during the last decades was in line with the general decreases observed at all sites along the Baltic (Miller et al. 2013; Soerensen and Faxneld 2022). At the end of the period analyzed in this study (2018), the three contaminant groups were below the thresholds set by the European Commission for environmental pollution hazards and human consumption. However, more stringent and updated thresholds have recently emerged for dioxins. In fact, in October 2022, the World Health Organization expert panel reevaluated toxic equivalency factors and calculated that updated TEQs limits values should be approximately halved for several relevant human food products compared when using TEQs established in 2005 to give an equivalent estimate (DeVito et al. 2024). This indicates that both thresholds and calculated TEQs for PCDD/Fs in fish should be revisited once the updated TEF system is formally adopted in regulatory frameworks. Furthermore, it has already been shown that higher concentrations are expected if skin or subcutaneous fat were included in the analyses (Aune et al. 2003; Miller et al. 2013) which is relevant not only for marine predators but also for human consumption since herring is indeed often consumed with skin. For these reasons, improving knowledge about causes driving the actual levels of contaminants in this keystone species within the Baltic ecosystem is crucial from both an ecological and a human health perspective.

While many studies have mentioned and discussed the role of emission reduction and related atmospheric deposition as the driver of contaminant concentration in Baltic Sea herring (Peltonen et al. 2007; Miller et al. 2013; Wiberg et al. 2013; Assefa et al. 2019), to our knowledge, this study is the first to explicitly address atmospheric deposition as a predictor. However, we found no influence of atmospheric deposition on contaminant burden in herring despite the significant atmospheric emission reductions undergoing in the period examined (Quaß et al. 2004; HELCOM 2020). Nevertheless, while it is common knowledge that atmospheric emission and subsequent deposition are the main diffuse pollution sources in the Baltic as a whole, other studies highlighted how non-atmospheric point sources could be significant on a local scale (Assefa et al. 2014). Contaminant release from sediments in industrialized/urbanized areas become increasingly important over time for coastal biota as a secondary pollution source (Verta et al. 2007; Sundqvist et al. 2009). In this regard, Assefa et al. (2018) found that the contribution of tetra-chlorophenol and penta-chlorophenol products from forestry-related industries in countries around the Baltic Sea had a greater-than-expected impact on dioxin concentrations in herring. Moreover, the ongoing effort in the creation/restoration of wetlands in the Baltic region may remobilize previously deposited mercury stored in the soil over the years (Gębka et al. 2019), inadvertently increasing mercury levels in surrounding water systems. Therefore, we cannot exclude the greater importance of contaminant input if all potential sources were included in the current analyses. However, it is unlikely that herring would be strongly influenced by local-scale sources, given the migratory nature of the species (Wiberg et al. 2013). Given the relative stability of contaminant concentrations despite emission reductions, it is not unreasonable to consider that other herring-related ecological factors, themselves influenced by environmental changes, may have had a greater contribution to the observed contaminant levels (Miller et al. 2013; Wiberg et al. 2013; Assefa et al. 2018).

Our results showed that alterations in herring trophic ecology, together with changes in the productivity base caused by nitrogen-fixing cyanobacterial blooms occurring over the past decades, were the primary factors influencing the concentrations of dioxins, PCBs, and mercury in herring in the study area (WGB). The positive relation between higher trophic ecology metrics—such as δ^15^N range, δ^13^C range, SEAb, and their lagged versions—with increased contaminant levels aligns with the principle that feeding across multiple trophic levels facilitates biomagnification of harmful substances, whereas reduced trophic diversity is associated with lower contaminant concentrations (Kiljunen et al. 2007). Adult herring such as those analyzed here feed both on lower trophic level zooplankton and amphipods and higher trophic level organisms such as mysids (Arrhenius and Hansson 1993; Möllmann et al. 2004). Overall, our findings suggest a general decrease in the trophic diversity of herring, which may have mitigated biomagnification in more recent times. Specifically, a decrease in trophic position and trophic diversity was evident from 1995 onwards. Interestingly, the PLSR analysis did not identify TP itself as a significant driver of contaminant burden. This could be due to the potential confounding effect of physiological stress (e.g., starvation or altered condition status) on TP value calculations. As stress can alter metabolic processes and thus isotope signatures, especially compound-specific isotope composition (Ek et al. 2018; Karlson et al. 2024), this may obscure the direct relationship between trophic position and contaminant burden. This apparently contradicts a previous finding for the Bothnian Sea herring from Wiberg et al. (2013) where a shift upward by approximately one trophic position over time was detected. However, as stated by the authors themselves, results from that study should be interpreted with caution since the trophic position was calculated from bulk isotope data in herring without an isotope baseline. The authors hypothesized a diet change from predominantly lower trophic level zooplankton to higher trophic level mysids which appears to be in line with a higher inter-specific competition with zooplanktivorous sprat and three-spined sticklebacks (Casini et al. 2010; Eklöf et al. 2020; Olin et al. 2022). Yet, this apparent contradiction aligns with basin-wise differences: while in the Bothnian Sea, a large proportion of higher trophic level organisms, such as mysids, are often seen in the herring diet (Parmanne et al. 2006); in the Baltic Sea Proper, herring of similar size to those analyzed in this study predominantly rely on the zooplankton (Arrhenius and Hansson 1993). Additionally, anoxic conditions in the Baltic Proper have reduced the habitat available for nektobenthic species, potentially limiting diet shift from zooplankton to nektobenthic prey (Kiljunen et al. 2020). In this regard, we found that zooplankton biomass was stable in the study area with a slight increase in mean size during the periods analyzed. This is most likely due to the slow recovery of intermediate-size copepods and large cladocerans from the decline that occurred in the mid-1990s. On the other hand, while no complete time series regarding the status of mysid populations is available for the area, comparing point data collected in 2008 with those from the 1980 s, Ogonowski et al. (2013) found a decrease in total mysid biomass by 50%. In line with these observations, PLSR results highlighted that dioxin burden is negatively associated with the zooplankton biomass, supporting the idea that a higher ratio of zooplankton-to-mysids in herring diet due to prey availability could actually reduce biomagnification. This pattern is not directly supported by the models for CB-153 and mercury; however, as discussed later, interactions between salinity and sprat competition, affecting herring–zooplankton dynamics, may have confounded and weakened these relationships. Another explanation could be that the trophic position of herring’s dominant prey assemblage has declined, rather than a significant change in the herring’s diet contribution itself. In a recent study, Karlson et al. (2024) claimed that the recent decrease in herring TP may reflect a diet dominated by lower TP zooplankton in comparison to the mid-1990s, where the sudden appearance of the invasive predatory zooplankton *Cercopagis pengoi* had led to a temporary but significant increase in TP values for young herring (Gorokhova et al. 2005). Furthermore, a shift in the mysid community towards species feeding at a lower trophic position has also been observed in the area (Ogonowski et al. 2013). However, to properly disentangle the relative importance of the two non-mutually exclusive phenomena, a longer time series of prey isotope data would be needed together with herring gut content analyses.

Besides biomagnification from diet, contaminant burden in herring and other organisms can change throughout their lifespan (Airaksinen et al. 2014). Considering the general decrease in the growth of Central Baltic herring over the last decades (Casini et al. 2010; ICES 2023), we expected higher age (longer time at sea) to be an important factor in counteracting the reduction of contaminants in herring expected from the ongoing emission reduction. However, we found that time spent at sea was of notable importance only in the PCDD/Fs model, where higher dioxin levels indeed were linked to older age. Despite this, age-dependent bioaccumulation appears to be of lower significance compared to the support for cyanobacteria-mediated dilution (FCA; details below). In the longer time series of mercury and CB-153, the effect appears to be minimal or even absent. Similarly, limited support for bioaccumulation in herring below 5 years has been found in the Northern Baltic Proper (Airaksinen et al. 2014). The limited variability observed in our data is likely due to the study’s narrow age range (3–5 years old), which aligns with the monitoring program’s intentional design to minimize biological variation by consistently selecting fish of similar size and age each year (Miller et al. 2013; Ammar et al. 2024), and further controlled through standardization of age by length (see the “Data pre-treatment and statistical analysis” section in the “Materials and methods” section). As proof of this, authors that combined younger individuals from the Marine Monitoring Program with external sampling, expanding the age span analyzed, found evidence of age-dependent dioxins bioaccumulation for Baltic herring (e.g., Soerensen et al. 2024). Therefore, while older and slower-growing fish can bioaccumulate (Peltonen et al. 2007), the biodilution mechanisms may play a bigger role in reducing contaminant concentrations, especially in younger individuals, as in this study. The lack of compelling evidence of high age-dependent bioaccumulation could also be due to increased reliance on preying on lower trophic level organisms (see above) and excretion of contaminants associated with gonads partition during yearly spawning events (Huynh et al. 2007; Frantzen et al. 2011). In this regard, seasonal changes in lipid reserves significantly affecting contaminant concentrations were observed in Baltic herring (Wiberg et al. 2013; Soerensen et al. 2024). This lipid cycling leads to increased wet-weight contaminant concentrations in spring, when lipid stores are depleted post-spawning, compared to lower and more stable concentrations in autumn (Bignert et al. 1993; Nyberg et al. 2015). In contrast, mercury, binding primarily to muscle proteins, is less directly influenced by seasonal lipid variation (Frantzen et al. 2015) but could still exhibit minor seasonal changes due to metabolic shifts and altered feeding rates between seasons.

Our study highlights how climate change-induced alterations on the productivity base may influence and drive contaminant burden in the Baltic biota. Previous studies focusing on benthic organisms have found that increased primary production from eutrophication can both increase contaminant uptake in deposit-feeders (Gunnarsson 2000) and reduce contaminant concentrations in filter-feeders (Ek et al. 2021). In addition to the well-known mechanisms of phytoabsorption sequestration (i.e., decrease of contaminant in the water phase due to sorption onto phytoplanktonic biomass; Koelmans et al. 2001; Lignell 1993) and bloom-induced dilution (i.e., decrease of the contaminant concentrations per unit of biomass due to adsorption onto larger phytoplanktonic biomass; Chen and Folt 2005), we hypothesized that N-fixing cyanobacterial bloom, which is favored in a warmer climate as well as by eutrophication (Kahru et al. 2020), could help in the reduction of contaminant burden in herring by supporting somatic growth biodilution (Simoneau et al. 2005). Even though several trophic levels separate nitrogen-fixation cyanobacteria and herring, N-fixing cyanobacterial summer blooms stimulate secondary production (Karlson et al. 2015; Svedén et al. 2016; Motwani et al. 2018), benefiting fish growth conditions (Taylor et al. under review), and therefore favoring biodilution. In our models, we found that all cyanobacteria-related proxies supported biodilution of the three contaminant groups analyzed (where higher values of the cyanobacteria-related proxies were generally associated with lower contaminant concentrations in fish). The apparently contrasting effect of cyanobacterial biovolume and FCA in the PCDD/Fs model was most likely due to differences in bloom characteristics and temporal dominance of the co-dominant cyanobacteria species. Specifically, FCA satellite data are good tracers for the more toxic blooms driven by *Nodularia spumigena*, a species that forms dense surface accumulations, while in situ collected cyanobacteria biovolume is more suitable to monitor *Aphanizomenon *sp., a non-toxic species that typically distributed deeper in the water column (HELCOM 2018; Kahru et al. 2020). There is evidence that the ratio of toxic/non-toxic species has increased in the whole Baltic Sea (Andersin et al. 2003). In particular, Figure S2 of the Supplementary Information shows that there has been a notable and continuous increase in *Nodularia spumigena* at the expense of *Aphanizomenon *sp. in the study area since the 1980 s until *Nodularia spumigena* became the dominant species in the open-sea station from 2010 onwards. For this reason, it is reasonable to think that the general decline in cyanobacteria biovolume in the water column in more recent times, not observed in FCA, is related to this. Furthermore, the deviation between these two cyanobacteria proxies could be in part related to spatio-temporal differences in how the data were collected and/or produced (e.g., lack of replicates in the monitoring program already highlighted by Rolff et al. 2007).

In this scenario, the increase in summer blooms over the last decades (Kahru et al. 2020) could most likely have locally supported an improvement in herring growth and reduced concentration of contaminants as a result of the three dilution mechanisms mentioned above. However, significant efforts have been made to reduce eutrophication and algal blooms, primarily by decreasing the levels of nutrients such as phosphorus and nitrogen entering the Baltic Sea (Gustafsson et al. 2012). While the reduction of nitrogen primarily impacts spring blooms (Graneli et al. 1990), a reduced input of phosphorus would decrease the summer bloom-forming cyanobacteria, consequently affecting secondary production and potentially leading to decreased fish production during summer (Hansson et al. 2007).

On the contrary, Steinkopf et al. (2024) showed that trophic lengthening in mesozooplankton driven by massive cyanobacterial blooms in coastal ecosystems may be carried over to fish, promoting biomagnification and potentially counteracting any dilutional effect. Moreover, it should be emphasized that high-intensity cyanobacterial blooms could be detrimental by creating toxic and anoxic conditions in the Baltic, the latter potentially promoting more methylation of mercury (Vahtera et al. 2007; Kumar et al. 2015) and directly affecting marine life health status (e.g., cod stock collapse; Köster et al. 2005). In this context, more toxic-dominated blooms (i.e., driven by *Nodularia spumigena*) are expected in the future due to rising temperatures, and higher precipitation and run-off (Gorokhova and Engstrom-Ost 2009) further exacerbating these negative ecological effects. Recognizing the ecological importance and dual effects of cyanobacteria, our study supports the need for careful nutrient input reductions and the establishment of ecological threshold levels for the dominant cyanobacteria. Such measures would aim to minimize harmful blooms without compromising the benefit that nitrogen fixation species could have on fish growth rates and the resultant accumulation of persistent contaminants in the fish.

The observed increases in temperature and salinity in the region aligned with the predicted trends for the Baltic area due to climate change, as described by Meier et al. (2022). Beyond potential direct effects on fish metabolism—though not considered in our models—both salinity and temperature are likely to impact contaminant burden through alterations in food web and algal bloom dynamics (see above). Specifically, salinity plays a crucial role in determining the structure and dynamics of the Baltic zooplankton community (particularly copepods; Gorokhova et al. 2016). Increased salinity generally supports higher zooplankton biomass, which subsequently affects herring diet and growth rates (Casini et al. 2008; Möllmann et al. 2009), thereby indirectly impacting contaminant bioaccumulation. However, the relationship between salinity, zooplankton dynamics, and herring growth is not linear but heavily affected by competition with sprat. At high population abundances (such as in the mid-1990s, when predation from cod was drastically reduced; Casini et al. 2008), sprat outcompetes herring for zooplankton resources and decouples herring growth from salinity regardless of the salinity condition (Casini et al. 2010). Although sprat dynamics were not explicitly considered in the analyses presented here, our results reflected the above-described hysteresis in the response of herring growth to salinity changes. Indeed, when models were constrained to periods of low sprat density (based on stock biomass data from Olin et al. 2022), contaminant concentrations in herring showed a negative correlation with salinity and zooplankton biomass (shorter dioxin time series). Conversely, in models that encompassed periods with higher sprat densities (longer CB-153 and mercury and time series), salinity exhibited no or even a positive effect on contaminant burdens (mercury and CB-153, respectively). These findings underscore the complexity of ecosystem interactions and the challenges in disentangling the effects of individual factors.

While the PLSR models presented and discussed here provide valuable insights into the drivers of contaminant variation in Baltic herring, several limitations should be acknowledged in interpreting these results. First, the relatively short time series available for PCDD/Fs restricts the model’s ability to capture long-term trends and inevitably reduces the robustness of inferred associations. Compared to the longer time series available for CB-153 and Hg, this temporal limitation introduces greater uncertainty in the final interpretation. Second, model stability and the interpretation of predictor associations are affected by several factors, including collinearity among predictors (though relatively low in this case), the relatively small sample size, and the use of geometric means to summarize yearly contaminant concentrations. While yearly means help improve model fit (e.g., *R*^2^*Y*), the variability in the data is not fully accounted for in the PLSR model output. For instance, in Fig. 3, the geometric mean concentration of CB-153 decreases each year from 1995 to 1998; however, due to high variability, these year-to-year differences are not statistically significant. Ultimately, although we included a set of relevant predictors supported by the literature, other potentially important variables not included here may have limited the explanatory power of the models. In fact, even if not explicitly accounted for in this study which focused more on bottom-up factors, top-down regulated phenomena such as recent shifts from predator to prey-driven ecosystem dynamics in the coastal community (Eklöf et al. 2020), the entry of new alien species in the community (Gorokhova et al. 2004; Hanson et al. 2020), and increasing trends of top predator population such as cormorants (Gagnon et al. 2015) are likely to indirectly impact the pathways through which contaminants bioaccumulate and biomagnify through the food web. Furthermore, the monitoring program sampling design, optimized to improve interannual comparability, limits biological variation (see above), potentially weakening the model’s ability to detect the complete contaminant-predictor relationships and limiting inference to specific fish life stages (e.g., 3–5-year-old herring).

Lastly, fishing pressure may also alter the levels of acquired contaminants in fish in various ways. For a stock exploited at a sustainable level, Peltonen et al. (2007) indicated that the average dioxin and PCB concentrations in catches would benefit from a higher exploitation rate, since commercially valuable herrings would on average be younger (i.e., shorter time at sea) and would therefore contain fewer contaminants. On the contrary, it is also proven that constant erosion of population structure in heavily exploited stocks drastically leads to a population composed mainly of slower-growing individuals (i.e., Rosa Lee effect, Kraak et al. 2019). The latter is more in line with the general finding of a slower growth rate for Baltic herring in the last decades (see above). Furthermore, even if in theory the effect of a higher exploitation rate on contaminant levels were positive, increasing the fishing pressure on an already overexploited stock such as Central Baltic herring (ICES 2023) would be ecologically deleterious and would violate any principle of fisheries sustainability and ecosystem-based management. Taken together, these limitations underscore the correlative nature of the PLSR approach (as well as other regression analyses), and we therefore urge caution against overinterpreting the results as evidence of causal relationships.

## Conclusion

The contaminant concentrations in fish such as herring are the result of several biological ecological, environmental, and anthropogenic drivers. The different drivers are often linked to each other by direct and indirect links (“feedback” effects) which could confound and mitigate the primary effect, making it more difficult to tease out actual contributions to contaminant levels in fish. While acknowledging the complexity of the topic, this study highlights that other factors beyond atmospheric emission/deposition may have influenced contaminant concentration in Baltic Sea herring. Among them, an altered productivity base in the form of N-fixing cyanobacterial summer blooms could support both somatic growth biodilution and phytoplankton bloom-induced dilution. It is therefore of primary importance to obtain a deeper understanding of the effects that a rapidly changing and developing ecosystem such as the Baltic Sea can have on measures implemented to minimize contaminant concentrations in fish. This would allow better support management decisions by taking into consideration all potential synergies or conflicts between the objectives of contaminants and nutrient input reduction, climate change, and fishing. Environmental monitoring programs of biota run by EU countries are of primary importance in order to follow temporal changes in harmful contaminants (Miller et al. 2013). In this view, the integration of archived biological samples from monitoring programs (i.e., ESB-SMNH) with additional specific analyses and ecological data as performed in this study could be a good practice to further enhance the scientific value of both national and international monitoring programs.

## Supplementary information

Below is the link to the electronic supplementary material.

ESM1 (DOCX 1.29 MB)

## Data Availability

Raw data sources and links are given in Table 2 of the current manuscript. Post-treatment data will be made available on request to authors.
