# Supplementary material for: Beyond emissions: unravelling the effects of ecosystem change on contaminant concentrations in herring from the Baltic Sea
Source: Environ Sci Pollut Res Int. 2025 Oct 2;32(40):22986–3008. doi: 10.1007/s11356-025-36988-y (PMC12553583; doi:10.1007/s11356-025-36988-y)
Supplement: Supplementary file 1 — (DOCX 1.29 MB) [file 11356_2025_36988_MOESM1_ESM.docx]

***Supplementary Information***

**Beyond emissions: Unravelling the effects of ecosystem change on contaminant concentrations in herring from the Baltic Sea**

Francesco Masnadi ^1^*, John M. Taylor ^1^, Johan Näslund^2^, Elisabeth Nyberg^2^, Andrius Garbaras^3^, Elena Gorokhova^4^, Agnes M.L. Karlson ^1,5^

^1^ Department of Ecology, Environment and Plant Sciences, Stockholm University, Stockholm, Sweden.

^2^ Swedish Environmental Protection Agency, Stockholm, Sweden.

^3^ Center for Physical Sciences and Technology, Vilnius, Lithuania.

^4^ Department of Environmental Science, Stockholm University, Stockholm, Sweden.

^5^ Stockholm University Baltic Sea Centre, Stockholm, Sweden.

*corresponding author: e-mail: [francesco.masnadi@su.se](mailto:francesco.masnadi@su.se)

| Congeners | % |
| --- | --- |
| HpCDD | 8.3 |
| HpCDF1 | 4.16 |
| HpCDF2 | 70.8 |
| HxCDD1 | 8.3 |
| HxCDD2 | 0 |
| HxCDD3 | 16.6 |
| HxCDF1 | 0 |
| HxCDF2 | 0 |
| HxCDF3 | 0 |
| HxCDF4 | 8.3 |
| OCDD | 8.3 |
| OCDF | 45.8 |
| PeCDD | 0 |
| PeCDF1 | 0 |
| PeCDF2 | 0 |
| TCDD | 12.5 |
| TCDF | 0 |

Table S1 – Table showing the percentage of values below the LOD for each PCDD/Fs congener.


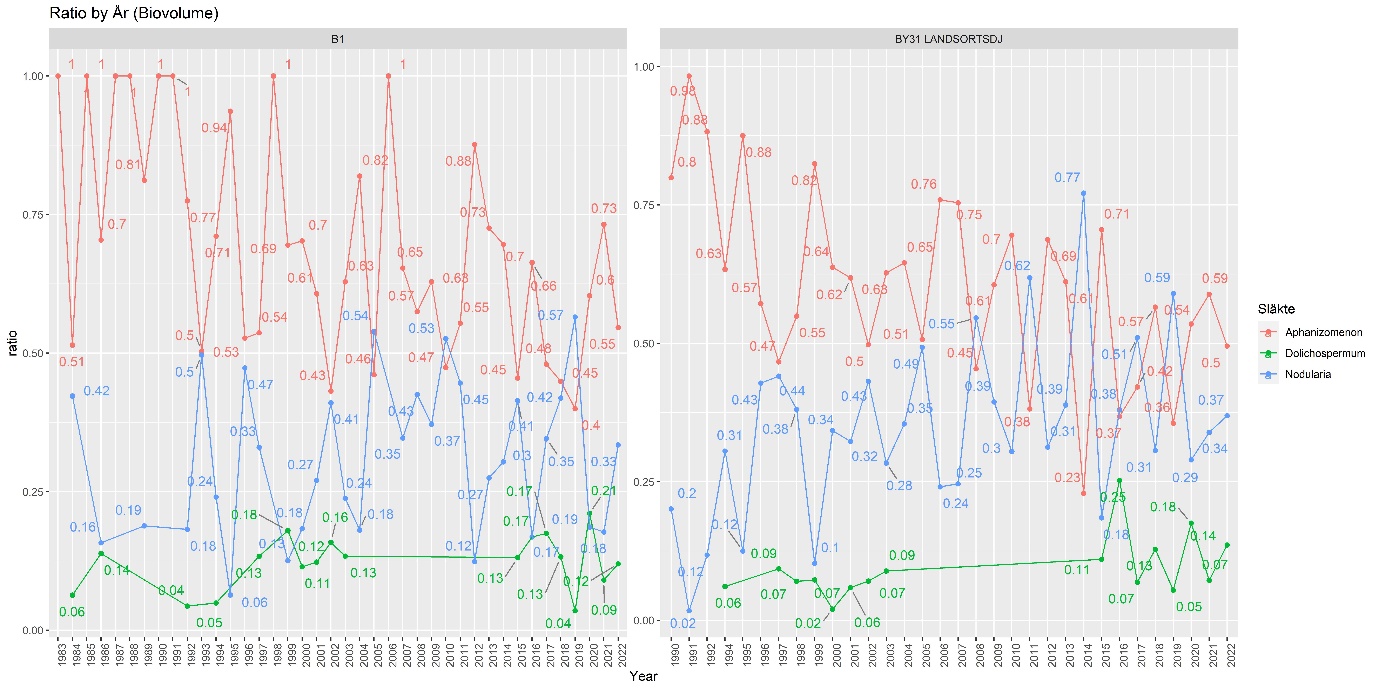


Fig. S2 – Timeseries of species composition (% on the total biovolume) of the three blooming N-fixing cyanobacteria species in the area: *Aphanizomenon sp.,* *Nodularia spumigena*, and *Dolichospermum spp*


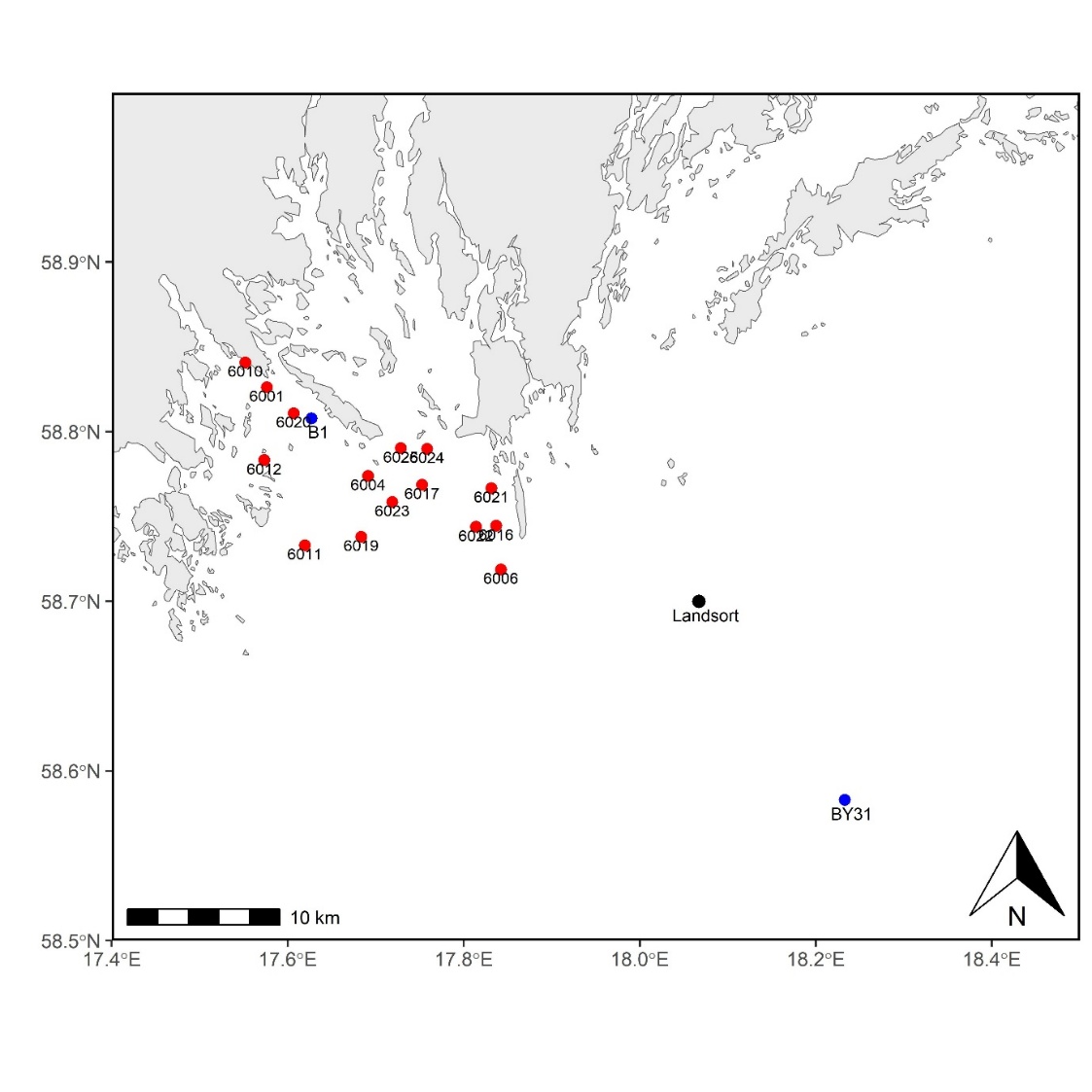


Fig. S3 – Zoomed-in map showing cyanobacteria and zooplankton stations (blue points) and benthos stations (red points) in the Askö area included the Swedish National Marine Monitoring Program. Landsort sampling station of the Swedish National Monitoring Program for Contaminants in Marine Biota (herring collection) is also shown for reference


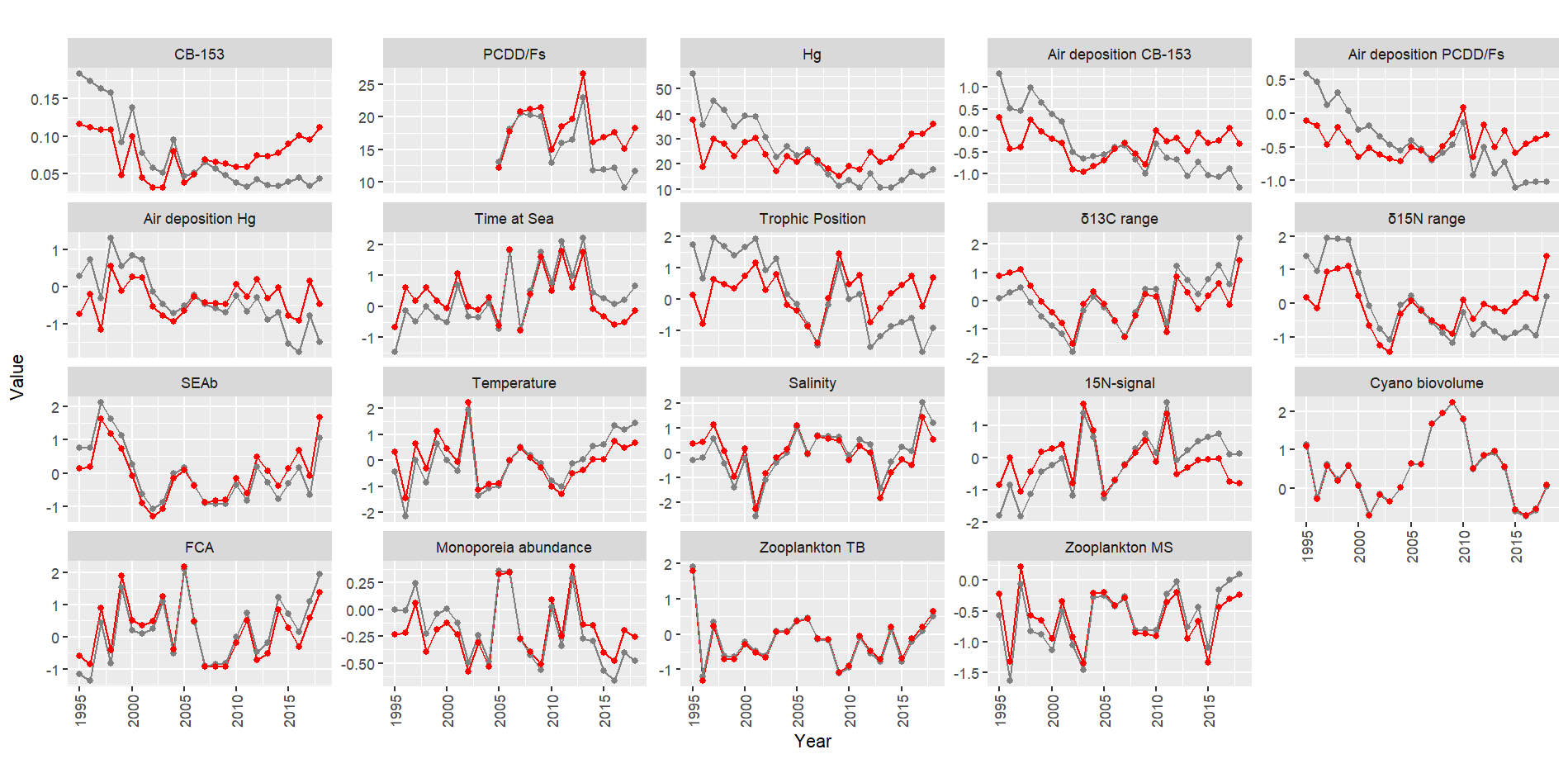


Fig. S4 – Year-detrended variables used as input data in PLSR analyses versus original values. Year-detrended values are the mathematical equivalent of using residuals from multiple independent linear regressions with year as the predictor. Grey lines = original values, red lines = year-detrended values. Detailed description of the variables in Table 1 and 2


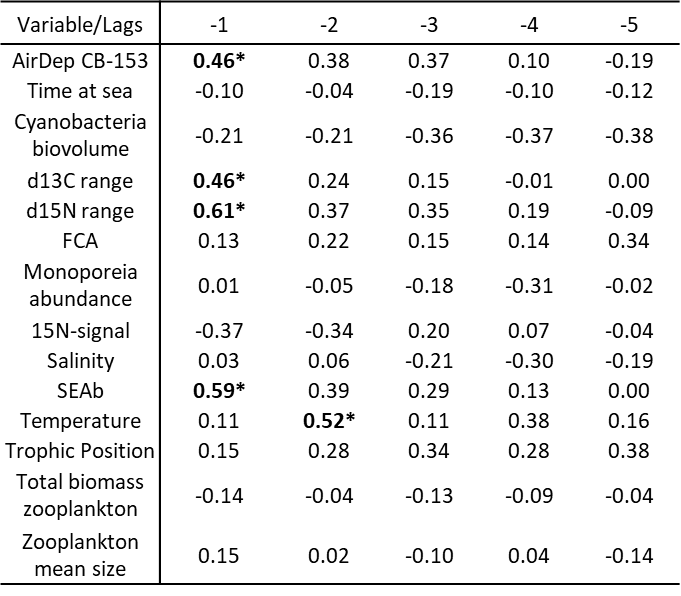


Table S5 - Cross-correlation analysis between CB-153 and predictor variables (max lag tested -5 years considering the life span of the fish sampled). *Significant Lags: lags where the cross-correlation is significantly different from zero (outside the confidence intervals). These are the lags at which the predictor series may have a meaningful impact on the CB-153 response series.


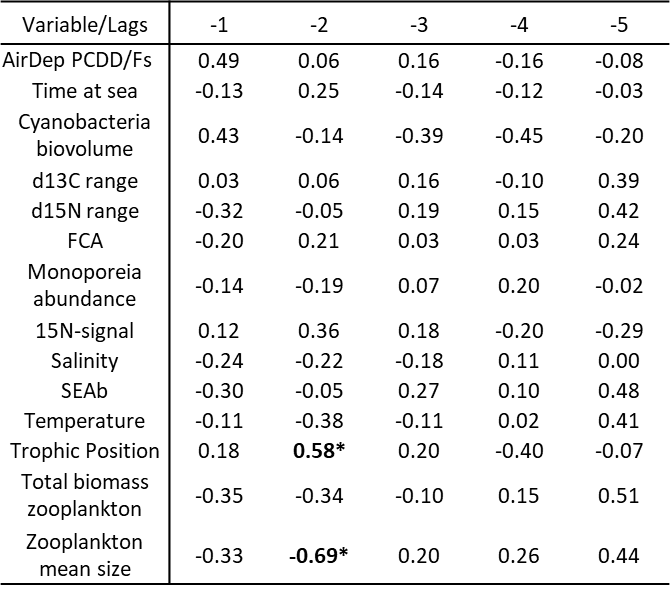


Table S6 - Cross-correlation analysis between PCDD/Fs and predictor variables (max lag tested -5 years considering the life span of the fish sampled). *Significant Lags: lags where the cross-correlation is significantly different from zero (outside the confidence intervals). These are the lags at which the predictor series may have a meaningful impact on the PCDD/Fs response series.


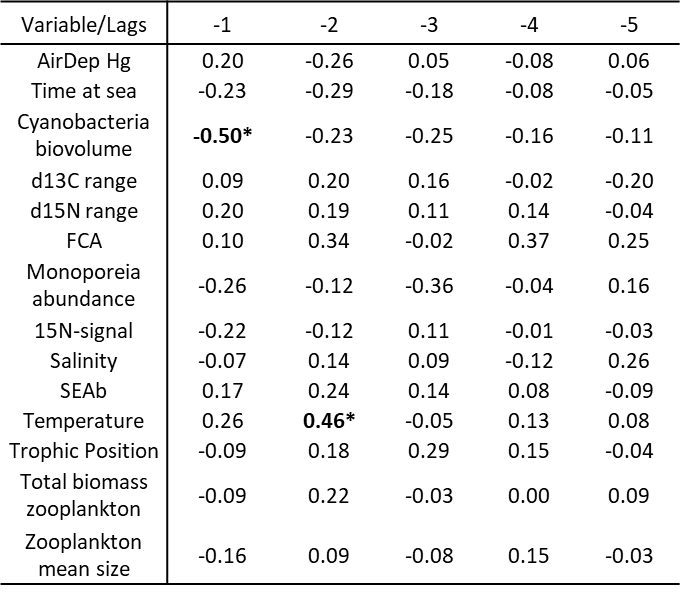


Table S7 - Cross-correlation analysis between Hg and predictor variables (max lag tested -5 years considering the life span of the fish sampled). *Significant Lags: lags where the cross-correlation is significantly different from zero (outside the confidence intervals). These are the lags at which the predictor series may have a meaningful impact on the Hg response series.


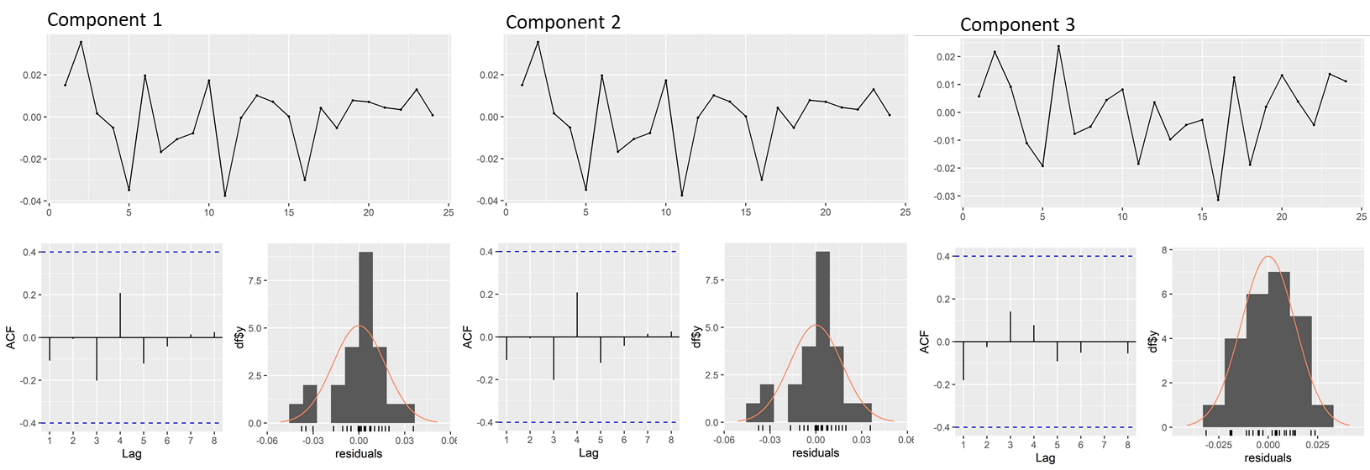


Fig. S8 – Time plots of the residuals, corresponding ACF, and residual histogram by model components for the CB-153 PLSR model. The time series plot of the residuals is provided to visually inspect for any patterns or trends. The ACF plot of the residuals is used to detect any autocorrelation (no autocorrelation when values fall within the 95% confidence intervals represented by the dotted blue lines). The histogram of the residuals is generated to evaluate the normality assumption


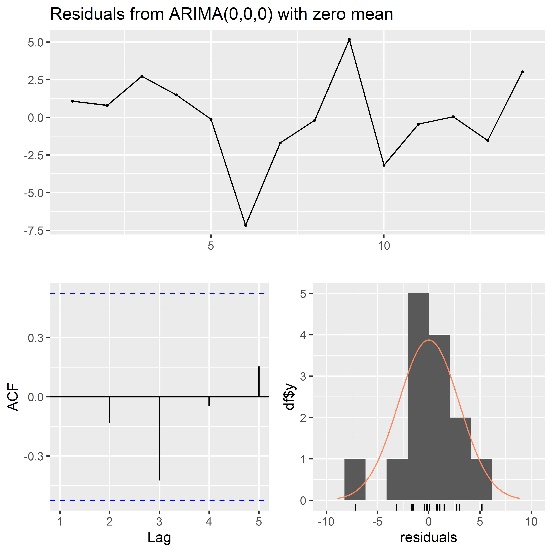


Fig. S9 – Time plot of the residuals, corresponding ACF, and residual histogram by model component (only one present) for the PCDD/Fs PLSR model. The time series plot of the residuals is provided to visually inspect for any patterns or trends. The ACF plot of the residuals is used to detect any autocorrelation (no autocorrelation when values fall within the 95% confidence intervals represented by the dotted blue lines). The histogram of the residuals is generated to evaluate the normality assumption


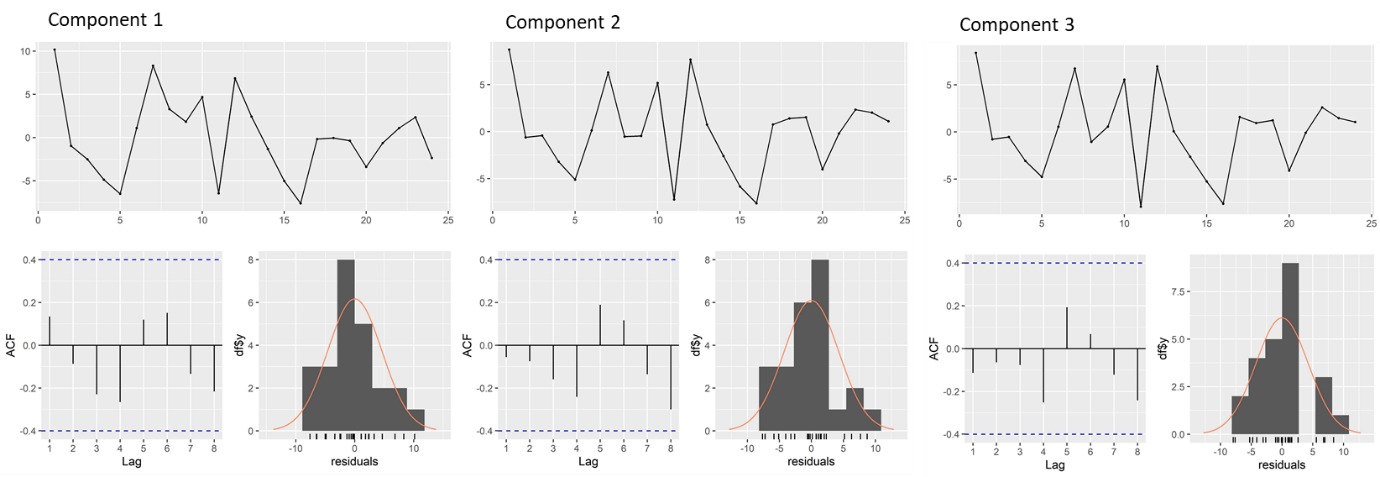
Fig. S10 – Time plot of the residuals, corresponding ACF, and residual histogram by model components for the Hg PLSR model. The time series plot of the residuals is provided to visually inspect for any patterns or trends. The ACF plot of the residuals is used to detect any autocorrelation (no autocorrelation when values fall within the 95% confidence intervals represented by the dotted blue lines). The histogram of the residuals is generated to evaluate the normality assumption

| Year | CB-153 | CI CB-153 lower | CI CB-153 upper | Hg | CI Hg lower | CI Hg upper | PCDD/Fs |
| --- | --- | --- | --- | --- | --- | --- | --- |
| 1995 | 0.184 | 0.131 | 0.258 | 55.997 | 42.893 | 73.104 |  |
| 1996 | 0.173 | 0.120 | 0.250 | 35.446 | 27.501 | 45.685 |  |
| 1997 | 0.164 | 0.121 | 0.223 | 45.188 | 32.533 | 62.766 |  |
| 1998 | 0.158 | 0.117 | 0.215 | 41.540 | 30.837 | 55.956 |  |
| 1999 | 0.092 | 0.069 | 0.122 | 35.080 | 24.181 | 50.891 |  |
| 2000 | 0.138 | 0.097 | 0.198 | 39.048 | 30.680 | 49.697 |  |
| 2001 | 0.077 | 0.058 | 0.102 | 38.982 | 29.097 | 52.225 |  |
| 2002 | 0.057 | 0.040 | 0.082 | 30.762 | 27.374 | 34.570 |  |
| 2003 | 0.051 | 0.037 | 0.070 | 22.699 | 18.587 | 27.721 |  |
| 2004 | 0.095 | 0.062 | 0.146 | 27.127 | 18.079 | 40.704 |  |
| 2005 | 0.047 | 0.039 | 0.057 | 23.287 | 18.911 | 28.676 | 13.026 |
| 2006 | 0.051 | 0.046 | 0.057 | 25.633 | 20.866 | 31.489 | 18.088 |
| 2007 | 0.066 | 0.040 | 0.108 | 20.516 | 12.869 | 32.708 | 20.477 |
| 2008 | 0.055 | 0.044 | 0.068 | 15.927 | 10.849 | 23.634 | 20.242 |
| 2009 | 0.048 | 0.038 | 0.061 | 11.339 | 8.829 | 14.561 | 20.009 |
| 2010 | 0.038 | 0.029 | 0.049 | 13.701 | 10.703 | 17.541 | 12.935 |
| 2011 | 0.032 | 0.027 | 0.038 | 10.711 | 8.984 | 12.769 | 15.941 |
| 2012 | 0.042 | 0.030 | 0.058 | 16.056 | 10.329 | 24.957 | 16.441 |
| 2013 | 0.035 | 0.027 | 0.044 | 10.478 | 8.261 | 13.291 | 22.889 |
| 2014 | 0.033 | 0.026 | 0.041 | 10.500 | 9.302 | 11.852 | 11.773 |
| 2015 | 0.039 |  |  | 13.672 |  |  | 11.955 |
| 2016 | 0.045 | 0.030 | 0.066 | 16.845 | 10.741 | 26.417 | 12.136 |
| 2017 | 0.033 | 0.024 | 0.045 | 15.332 | 9.479 | 24.798 | 9.082 |
| 2018 | 0.043 | 0.027 | 0.069 | 17.741 | 11.843 | 26.577 | 11.625 |

Table S11 – Annual geometric mean concentrations of contaminants in herring from the Landsort station (Western Gotland Basin), as presented in Figure 3 of the main text. The 95% Confidence interval is calculated for CB-153 and Hg where individual-level samples were available*.* The 2015 value was interpolated; confidence intervals are not reported for that year. CB-153: μg/g lipid weight; Hg: ng/g wet weight; PCDD/Fs: pg TEQ/g lipid weight


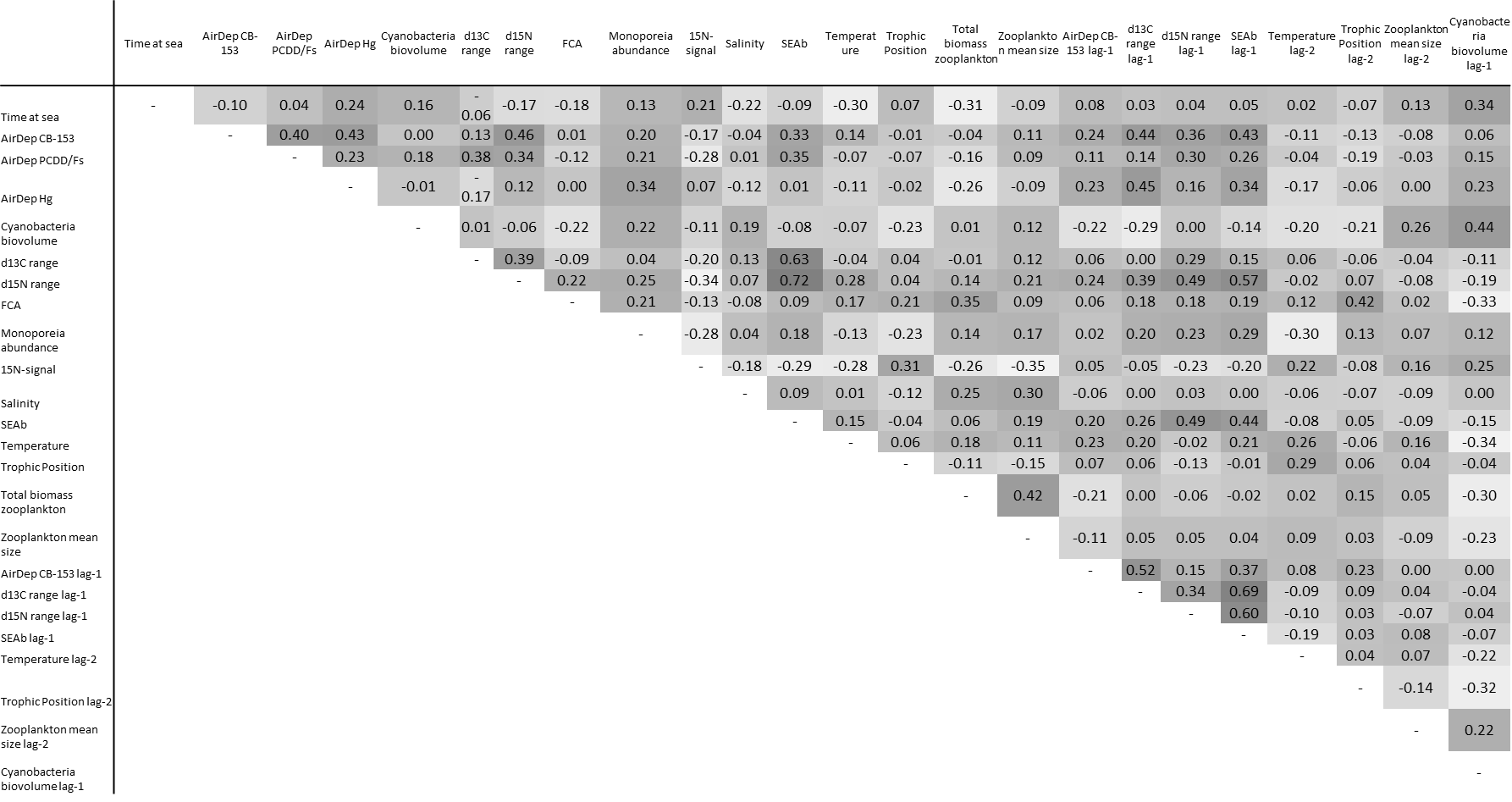


Table S12 – Correlation matrix plot showing pairwise correlation coefficients between candidate predictors. Time series 1995 – 2018. Detailed description of the predictors in Table 2


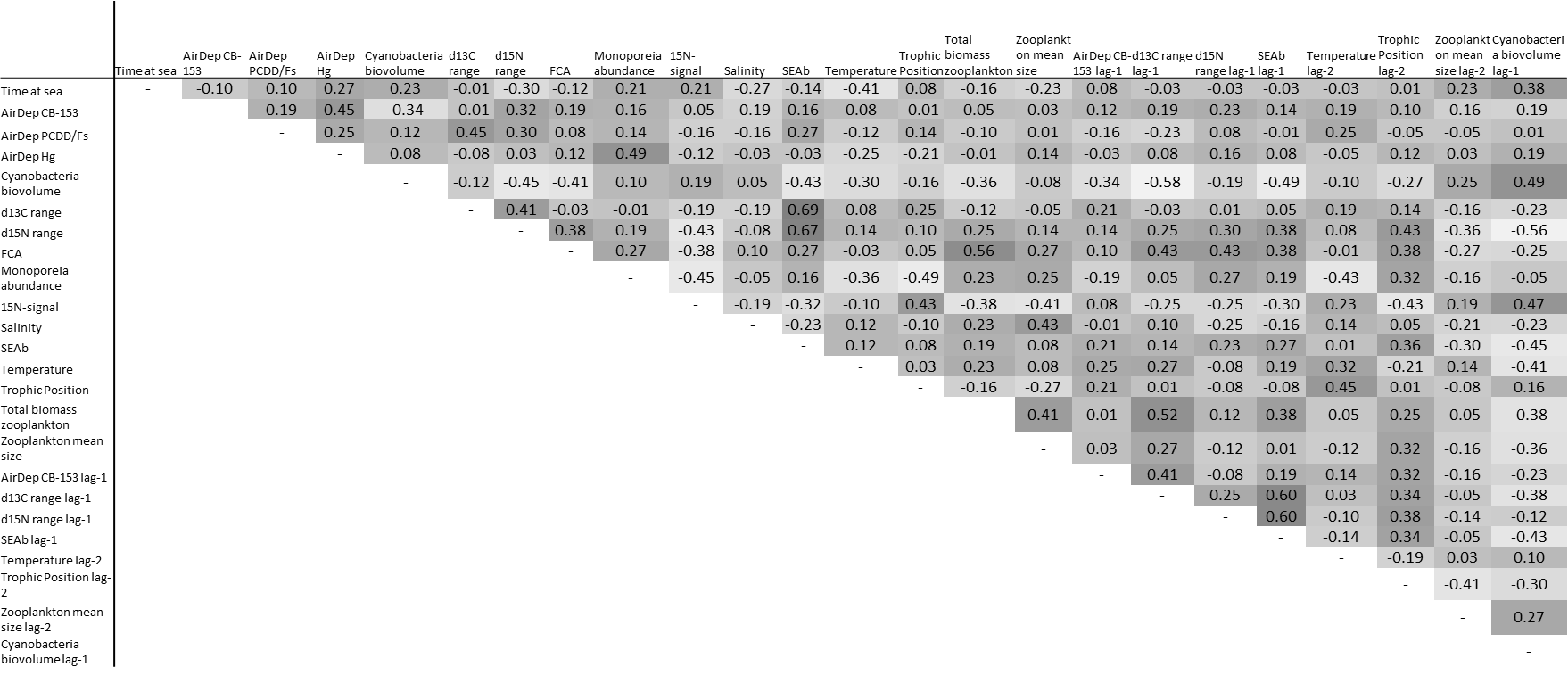


Table S13 – Correlation matrix plots showing pairwise correlation coefficients between candidate predictors. Time series 2005 – 2018. Detailed description of the predictors in Table 2


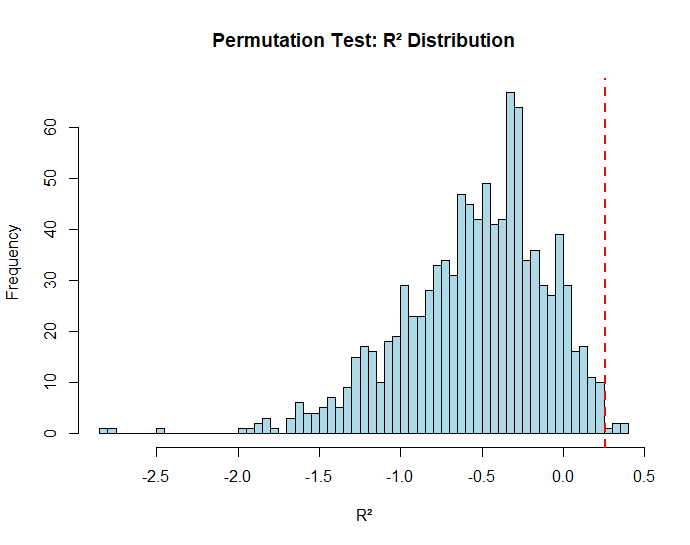


Fig. S14 – Comparison of PCDD/Fs model with a random permuted data version to assess whether the original model performs significantly better than chance. The response variable was randomly shuffled and the model was re-fitted multiple times (n=1000) to compare the original R^2^Q (0.26; red dotted line) with the distribution of permuted R^2^Q values. Significance of the test was checked by permutation p-values (0.004; proportion of permuted models with R^2^Q >= original R^2^Q). A p-value of 0.004 indicates that the original model performs significantly better than the random (permuted) one

Below the code used for PLSR analyses (based on *pls* R package ver. 2.8-3. November 2023):

##################################################################################

# VIP function from <https://mevik.net/work/software/VIP.R>;

# Copyright © 2006.2007 Bjørn-Helge Mevik

VIP <- function(object) {

if (object$method != "oscorespls")

stop("Only implemented for orthogonal scores algorithm. Refit with 'method = \"oscorespls\"'")

if (nrow(object$Yloadings) > 1)

stop("Only implemented for single-response models")

SS <- c(object$Yloadings)^2 * colSums(object$scores^2)

Wnorm2 <- colSums(object$loading.weights^2)

SSW <- sweep(object$loading.weights^2. 2. SS / Wnorm2. "*")

sqrt(nrow(SSW) * apply(SSW. 1. cumsum) / cumsum(SS))

}

# regr.eq is a function which gives the regression coefficients

regr.eq <- function(model){

b <- model$Yloadings[1. ]

#Number of parameters in model

p <- ncol(model$model) - 1

# matrix for the loadings

t <- matrix(ncol = length(b).

nrow = p)

for(i in 1:length(b)){

t[. i] <- model$loadings[. i]

}

#Empty vector to be filled

foobar <- c()

#compute values

for(i in 1:p){

value <- t[i. ] * b

foobar[i] <- sum(value)

}

ret.data <- data.frame(VARIABLES = names(model$model)[-1].

REG.COEF = foobar)

return(ret.data)

}

# Writing the model formula

library(pls)

attach(db_PLSR)

y <- `response` # response: CB-13. PCDD/Fs or Hg

X <- data.frame(db_PLSR[10:23]) # selection of predictors

init.pred <- names(X)

# Fit PLSR model

X.frmla <- paste(c(names(X)). sep = "")

frmla <- as.formula(paste("y ~ ". paste(X.frmla. collapse = "+")))

# Initial model

model0 <- plsr(frmla. data = X. method = "oscorespls". validation = "LOO". scale = F)#(oscorespls aka the NIPALS algorithm).

# Select minimum component

rmsep_vals <- RMSEP(model0)

rmse_values <- rmsep_vals$val[1.1.-1] # remove intercept-only (0 components)

min.comp <- which.min(rmse_values) # select the ncomp that gives lower RMSEP

# Rerun the model with updated min.comp

model <- plsr(frmla. data = X. method = "oscorespls". validation = "LOO". scale = F. ncomp = min.comp)

summary(model)

#VIP.score <- VIP(model) # when only 1 comp

VIP.score <- VIP(model)[model$ncomp. ]

coef.size <- abs(coef(model. ncomp = model$ncomp))

VIP.limit <- 0.70

plot(y = VIP.score. x = coef.size. ylab = "VIP-scores". xlab = "Coefficient sizes")

text(y = VIP.score. x = coef.size. labels = names(X))

abline(h = VIP.limit. lty = 2)

# Variable selection based on VIP value

ind.2 <- which((VIP.score <= VIP.limit)) # remove variable according to VIP

# Refitting the model

X.upd <- X[. -ind.2]

X.frmla.upd <- paste(c(names(X.upd)). sep = "")

frmla.upd <- as.formula(paste("y ~ ". paste(X.frmla.upd. collapse = "+")))

u.model <- plsr(frmla.upd. data = X. method = "oscorespls". validation = "LOO".

scale = F. ncomp = min.comp)

# ARIMA: Time plots of the residuals. corresponding ACF. and residual histogram by model components

residuals_plsr <- residuals(u.model)

for (i in 1:dim(residuals_plsr)[3]) {

residuals_component <- residuals_plsr[. .i]

arima_model <- auto.arima(residuals_component)

print(summary(arima_model))

checkresiduals(arima_model)

title(main = paste("Residual Diagnostics for Component". i))

}

# R2Y is the explanatory capacity coefficient. R2Q is the prediction capacity coefficient

R2Q <- R2(u.model. estimate = "CV")$val[..min.comp+1] # at least > 0.4

R2Y <- R2(u.model. estimate = "train")$val[..min.comp+1] # at least > 0.7

regr.coef <- regr.eq(u.model) #regression coefficient

# Calculate SSW. Squaring the weightings and summing across components. then taking the square root

SSW <- sqrt(rowSums(abs(u.model$loading.weights)^2))

regr.coef$WRC <- abs(as.data.frame(SSW)$SSW*regr.coef$REG.COEF)

regr.coef$Effect_Direction <- ifelse(regr.coef$REG.COEF < 0 ."Negative" . "Positive" )

##################################################################################
